# Development of an assay system for the analysis of host RISC activity in the presence of a potyvirus RNA silencing suppressor, HC-Pro

**DOI:** 10.1186/s12985-022-01956-2

**Published:** 2023-01-17

**Authors:** Syuan-Fei Hong, Ru-Ying Fang, Wei-Lun Wei, Supidcha Jirawitchalert, Zhao-Jun Pan, Yu-Ling Hung, Thanh Ha Pham, Yen-Hsin Chiu, Tang-Long Shen, Chien-Kang Huang, Shih-Shun Lin

**Affiliations:** 1 Institute of Biotechnology, National Taiwan University, Taipei, 106 Taiwan; 2 Seed Improvement and Propagation Station, Council of Agriculture, Taichung, 427 Taiwan; 3 Department of Plant Pathology and Microbiology, National Taiwan University, Taipei, 106 Taiwan; 4 Department of Computer Science and Information Engineering, National Taiwan University, Taipei, 106 Taiwan; 5 Agricultural Biotechnology Research Center, Academia Sinica, Taipei, 115 Taiwan; 6 Center of Biotechnology, National Taiwan University, Taipei, 106 Taiwan

**Keywords:** AGO1 degradation, HC-pro, In vitro RISC assay, T-DNA insertion

## Abstract

**Background:**

To investigate the mechanism of RNA silencing suppression, the genetic transformation of viral suppressors of RNA silencing (VSRs) in Arabidopsis integrates ectopic VSR expression at steady state, which overcomes the VSR variations caused by different virus infections or limitations of host range. Moreover, identifying the insertion of the transgenic VSR gene is necessary to establish a model transgenic plant for the functional study of VSR.

**Methods:**

Developing an endogenous AGO1-based in vitro RNA-inducing silencing complex (RISC) assay prompts further investigation into VSR-mediated suppression. Three P1/HC-Pro plants from turnip mosaic virus (TuMV) (*P1/HC-Pro*^*Tu*^), zucchini yellow mosaic virus (ZYMV) (*P1/HC-Pro*^*Zy*^), and tobacco etch virus (TEV) (*P1/HC-Pro*^*Te*^) were identified by T-DNA Finder and used as materials for investigations of the RISC cleavage efficiency.

**Results:**

Our results indicated that the *P1/HC-Pro*^*Tu*^ plant has slightly lower RISC activity than *P1/HC-Pro*^*Zy*^ plants. In addition, the phenomena are consistent with those observed in TuMV-infected Arabidopsis plants, which implies that HC-Pro^Tu^ could directly interfere with RISC activity.

**Conclusions:**

In this study, we demonstrated the application of various plant materials in an in vitro RISC assay of VSR-mediated RNA silencing suppression.

**Supplementary Information:**

The online version contains supplementary material available at 10.1186/s12985-022-01956-2.

## Background

P1/HC-Pro fusion protein of potyvirus is the first identified viral suppressor of RNA silencing (VSR). The HC-Pro inhibits short-interfering RNA (siRNA) and microRNA (miRNA) regulatory pathways [1, 2]. Several studies have demonstrated that P1 enhances the suppression ability of the HC-Pro [3, 4]. Thus, the P1/HC-Pro fusion protein has been used to investigate the mechanism of silencing suppression [3]. The fusion protein can be cleaved by P1 protease activity in vivo to generate two separated P1 and HC-Pro proteins for adapting to potyvirus infection [5]. Several studies investigated the HC-Pro function through viral infection [6–9]. However, the unequal viral titers caused variation in HC-Pro amounts, which is challenging to evaluate the suppression efficiency. For instance, TuGK, a mild strain of turnip mosaic virus (TuMV) carrying the *GFP* gene, showed a lower viral titer than the severe strain TuGR (a wild-type TuMV carrying a *GFP* gene) [8]. Thus, the unequal viral titers between TuGK and TuGR result in different amounts of HC-Pros [8]. Moreover, different species viruses might have various host ranges, which limits the comparison of various HC-Pros on the same plant host. For instance, the zucchini yellow mosaic virus (ZYMV) cannot infect cruciferous plants. In contrast, TuMV cannot infect cucurbit species, which means the ZYMV HC-Pro (HC-Pro^Zy^) and TuMV HC-Pro (HC-Pro^Tu^) cannot be compared the suppression efficiency through the viral infection.

To overcome the titer variation and host range limitation, the ectopic expression of HC-Pros in various species using a transgenic plant approach is a good solution. Wu et al. ectopically expressed wild-type HC-Pro^Zy^ in Arabidopsis (*P1/HC-Pro*^*Zy*^ plant) to identify the critical amino acids on the FRNK motif of HC-Pro that are involved in VSR function [7]. Kung et al. and Sanobar et al. created transgenic Arabidopsis plants expressing the *P1/HC-Pro* gene of TuGR (*P1/HC-Pro*^*Tu*^ plant) or TuGK (*P1/HC-Pro*^*Tu−K*^ plant), which exhibit comparable amounts of HC-Pro, and demonstrated that the Arg of the FRNK motif of HC-Pro^Tu^ is critical for physical interaction with HEN1 (a methyltransferase for miRNA methylation) to result in HEN1 inhibition [8, 10]. Therefore, approximately 50% of unmethylated miRNAs exist in the cytoplasmic of *P1/HC-Pro*^*Tu*^ plants [10]. Surprisingly, Wei et al. demonstrated that inside of AGO1 from *P1/HC-Pro*^*Tu*^ plants only contain methylated miRNAs [11]. The *P1/HC-Pro*^*Tu−K*^ plant expressing an Arg182Lys mutation (FKNK) of HC-Pro (HC-Pro^Tu−K^), resulting in a loss 40% of the suppression ability in RNA silencing [8]. Hu et al. also demonstrated that *P1/HC-Pro*^*Tu*^ plants have lower AGO1 levels than transgenic Arabidopsis expressing the *P1/HC-Pro* gene of tobacco etch virus (TEV) (*P1/HC-Pro*^*Te*^ plant) and *P1/HC-Pro*^*Zy*^ plants; however, three P1/HC-Pro plants exhibit a common severe serrated-curled leaf phenotype, suggesting that various HC-Pros exhibit different ability in suppressing RNA silencing [4].

Many studies imply that interfering with RNA-induced silencing complex (RISC) assembly triggers autophagy for AGO1 degradation [12–14]. To further apply gene-silencing components or autophagy mutants to the other P1/HC-Pro plants, these T-DNA insertion positions should also be identified. T-DNA of *P1/HC-Pro*^*Tu*^ gene was inserted in chromosome 1 (at the 28,814,559^th^ nt) [15], and the *ATG8a* (an autophagy gene) is located on chromosome 4. Wei et al. and Shang demonstrated *ATG8a* knockout mutant in a *P1/HC-Pro*^*Tu*^ background (*P1/HC-Pro*^*Tu*^*/atg8a*^*ge*^ plants) restores the AGO1 levels to the level near those found in Col-0 plants, which indicates that HC-Pro^Tu^ triggers autophagic AGO1 degradation [11, 16].

Investigating the RISC activity during TuMV infection is a novel undertaking. Many studies have demonstrated in vitro RISC activity assays using Flag-tagged *AGO1* transgenic plant, which was generated in the *ago1-36* background [17, 18]. However, applying Flag-tagged AGO1 transgenic plants limits the RISC assay to plants with mutations in RNA silencing or these P1/HC-Pro plants. For instance, fifty-six different mutant alleles of *AGO1* have been identified with exhibit losses of particular miRNA regulatory functions [19]. If we want to study particular functions for those *ago1* mutants, we have to individually construct the Flag-tag on each mutated *AGO1* gene and transfer them into the *ago1-36* allele. Therefore, the best way is to generate an α-AGO1 antibody for endogenous AGO1 immunoprecipitation (AGO1-IP). Furthermore, Dicer-like 1 (DCL1) responds to miRNA biogenesis, whereas DCL2, DCL3, and DCL4 play roles in short-interfering RNA (siRNA) generation [20]. RNA-dependent-RNA polymerases (RDR2 and RDR6), HYPONASTIC LEAVES1 (HYL1), SERRATE (SE), and other AGOs (*e.g.*, AGO2, AGO4, and AGO7), which are involved in small RNA regulatory pathways or virus defense. These RNA silencing components also have related mutant lines that can be used as plant materials for studies of their RISC activity [9, 21–23]. Therefore, an α-AGO1 antibody that specifically immunoprecipitates endogenous AGO1 from any Arabidopsis material could help researchers further investigate gene silencing.

In this study, we identified the T-DNA insertion in various P1/HC-Pro plants to provide genetic information that allows us to generate more plant materials for the in vitro RISC assay. We also developed an α-AGO1 antibody for the in vitro RISC assay to evaluate RISC activity in these *P1/HC-Pr*o and virus-infected plants. We demonstrated that various HC-Pros of viral species have different RISC inhibition abilities, which opens a new field for further investigation of RNA silencing and suppression.

## Results

### Endogenous AGO1 IP for the in vitro RISC assay

By using endogenous AGO1, the in vitro RISC assay can eliminate the limitation of plant material; however, a good-quality α-AGO1 antibody that does not affect AGO1 function would determine whether the in vitro RISC assay can be successfully performed. Although commercial AGO1 is available, we made a homemade α-AGO1 antibody to pull down a large amount of AGO1 for the assay. Moreover, the generation knowledge can be applied to producing other AGO antibodies, *e.g.*, AGO2.

**Fig. 1:**
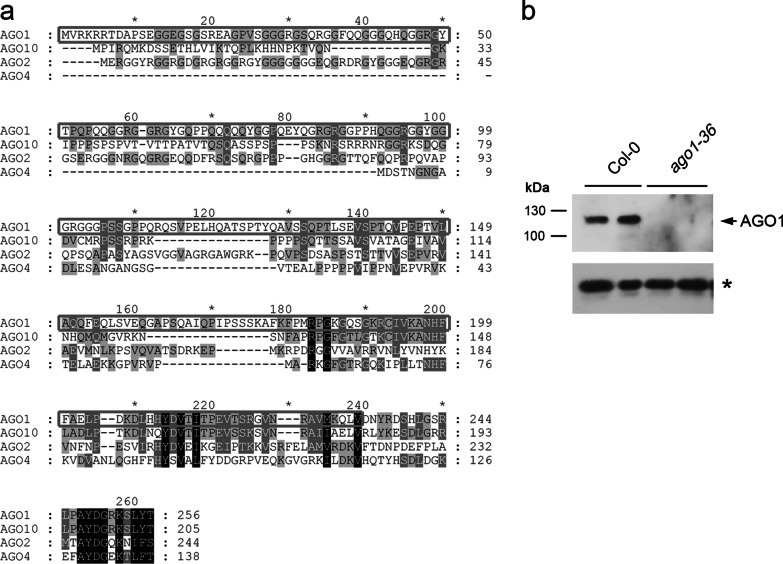
α-AGO1 antibody generation. **a** Amino acid alignment of the N-termini of AGO1, AGO2, AGO4, and AGO10. The gray box indicates the N-terminal region of AGO1 for antigen. **b** Evaluation of the α-AGO1 antibody for detection of endogenous AGO1 in Col-0 plants. The null *ago1-36* mutant serves as the negative control. The asterisk indicates tubulin, which was used as a loading control

The amino acid alignments of AGO1, AGO2, AGO4, and AGO10 showed that the 1-to-256-aa region is variable, with 12.2%-30.5% identity (Fig. 1a). Thus, the N-terminus of AGO1 (1–240 aa) was selected for recombinant protein production and purified as an antigen to the AGO1 antibody (Fig. 1a, box). Western blot analysis showed that the α-AGO1 antibody could recognize endogenous AGO1 (117 kDa) in Col-0 plants. In contrast, the AGO1 signal was absent in the *ago1-36* null mutant, suggesting that the homemade α-AGO1 antibody can be tested for its IP ability with an in vitro RISC assay (Fig. 1b).

**Fig. 2:**
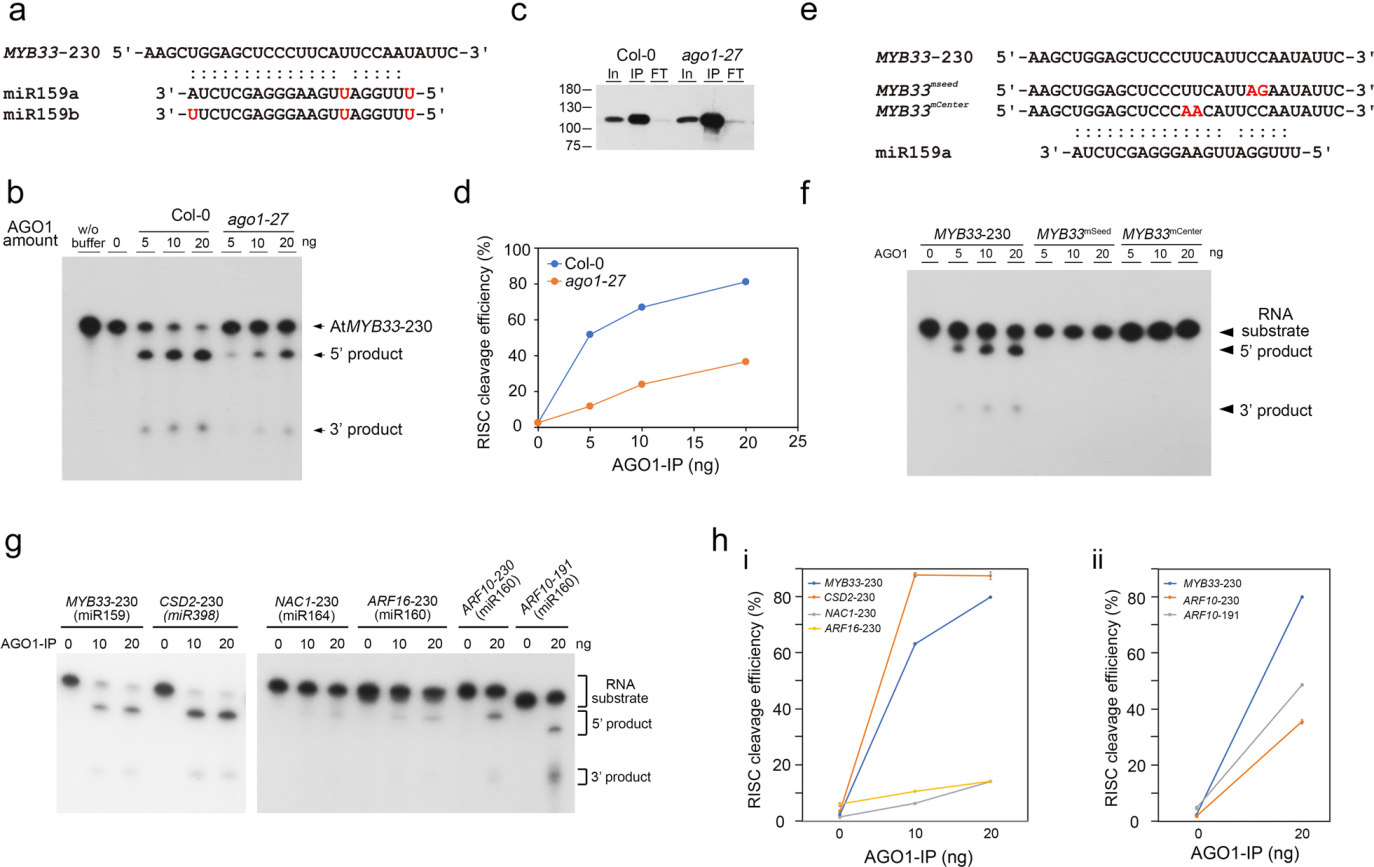
Establishment of an in vitro RISC assay. **a** Sequence pairing of *MYB33* RNA and miR159 isoforms. The mismatched nucleotides are highlighted in red. **b** The endogenous AGO1-IP products from Col-0 plant and *ago1-27* mutant were incubated with *MYB33*-230 RNA substrate to perform an in vitro RISC assay. The RNA substrate and cleaved RNA fragments are indicated with arrows. **c** The AGO1-IP amounts from Col-0 plant and *ago1-27* mutant were evaluated by western blotting with α-AGO1 IgG for normalization of the RISC cleavage efficiency. In: input; IP: immunoprecipitation; FT: flow through. **d** Normalized RISC cleavage efficiency for AGO1-IP from Col-0 plant and *ago1-27* mutant. **e** Sequencing pairing between miR159a and the *MYB33*^*mSeed*^ or *MYB33*^*mCenter*^. The mismatched nucleotides are highlighted in red. **f** In vitro RISC assay for *MYB33*-230, *MYB33*^*mSeed*^, and *MYB33*^*mCenter*^ RNA substrates. The RNA substrate and cleaved RNA fragments are indicated with arrows. **(g)** In vitro RISC assay for various miRNA-target substrates. **h** Normalized RISC cleavage efficiency for *CSD*-230, *NAC1*-230, and *ARF16*-230 RNA substrates (i). Normalized RISC cleavage efficiency for *ARF10*-230 and *ARF10*-191 RNA substrates (ii). The *MYB33*-230 RNA substrate served as a positive control

To evaluate the in vitro RISC assay with the α-AGO1 antibody, endogenous AGO1 was immunoprecipitated from Col-0 seedlings and *ago1-27* mutants and assessed with the RNA substrate *MYB33* (*MYB33*-230), which contained a target site for miR159a and miR159b (Fig. 2a). The AGO1-IP from Col-0 seedlings can target and cleave *MYB33*-230 (Fig. 2b). The signals of 5'- and 3'-cleaved fragments were increased when the amounts of AGO1-IP were increased, suggesting that AGO1 cleavage is dose-dependent (Fig. 2b). The *ago1-27* mutant, which exhibits an Ala992Val weak allele, showed attenuated slicer activity [24]. AGO1-IP from the *ago1-27* mutant revealed inefficient *MYB33*-230 cleavage (Fig. 2b). The amounts of AGO1-IP from Col-0 plants and *ago1-27* mutants were comparable (Fig. 2c). The 20 ng AGO1-IP from Col-0 plants resulted in 81% cleavage efficiency, whereas only 37% cleavage efficiency was observed with 20 ng AGO1-IP from *ago1-27* mutants (Fig. 2d). We further mutated the target site in the seed region (*MYB33*^*mSeed*^) and center region (*MYB33*^*mCenter*^) (Fig. 2e). The results showed no RISC cleavage of *MYB33*^*mSeed*^ or *MYB33*^*mCenter*^ substrates, whereas *MYB33*-230 was cleaved as a positive control (Fig. 2f). These results indicated that the α-AGO1 antibody could be used for the in vitro RISC assay.

Next, we also examined different RNA substrates, including *CSD2*-230 (miR398 target), *NAC1*-230 (miR164 target), *ARF16*-230 (miR160 target), and *ARF10*-230 (miR160 targets) (Fig. 2g). All RNA substrates exhibit different levels of cleavage efficiency. For example, *MYB33*-230 and *CSD2*-230 have higher cleavage efficiency than *NAC1*-230, *ARF16*-230, and *ARF10*-230 (Fig. 2h, panel i). We designed two *ARF10* substrates with 3'-end of different lengths: *ARF10*-230 (230 nt) and *ARF10*-191 (191 nt) (Fig. 2g). The data indicated that *ARF10*-191 exhibited better cleavage efficiency than *ARF10*-230 (Fig. 2h, panel ii). Thus, we assumed that the secondary structure of the RNA substrate and abundance of miRNA in AGO1 might affect the cleavage efficiency.

### Evaluating endogenous RISC activity in P1/HC-Pro plants

**Fig. 3:**
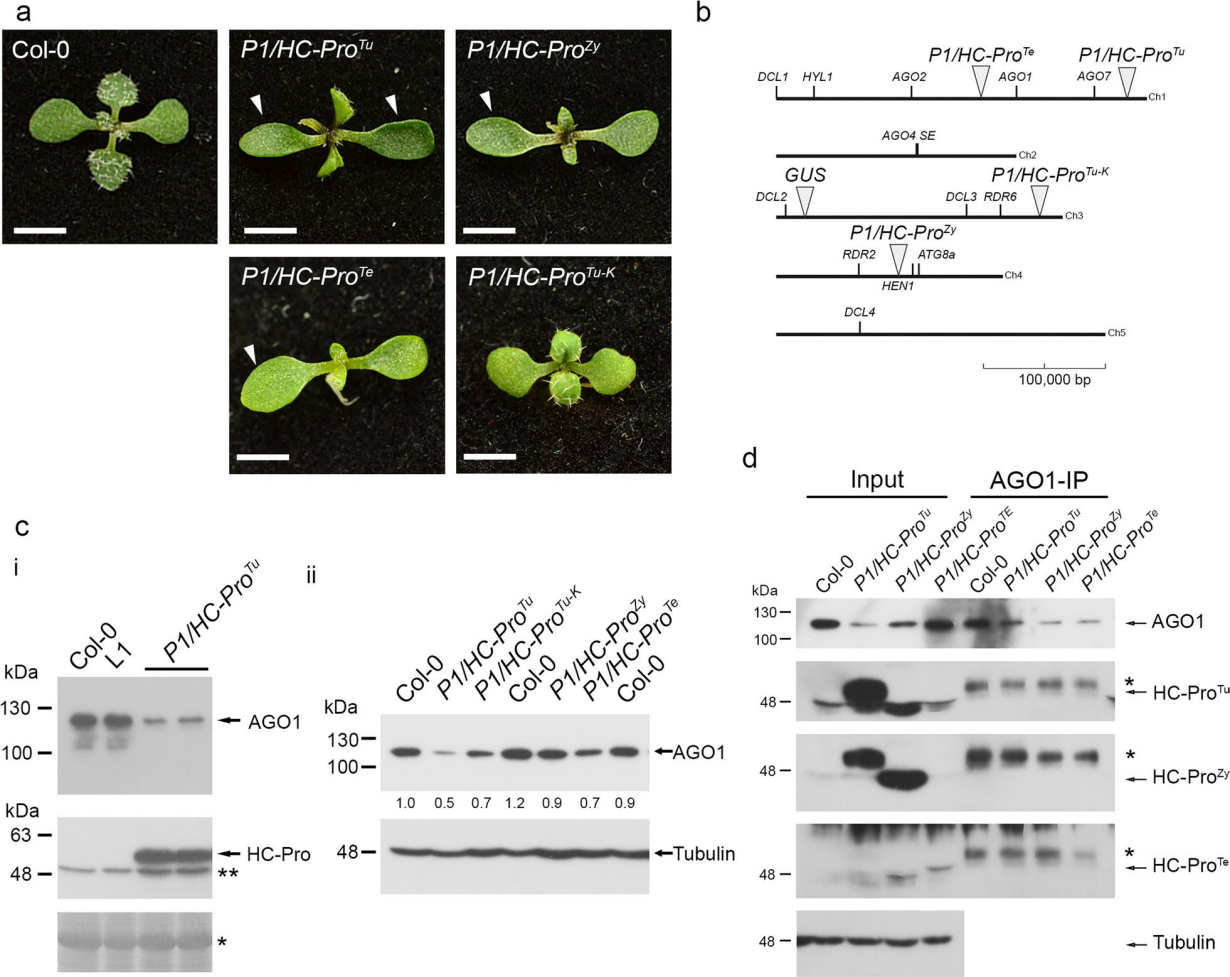
The functional studies of HC-Pro for AGO1 regulation. **a** Phenotypes of P1/HC-Pro plants. The photographs were obtained from 14-day-old seedlings. Bar, 0.2 cm. **b** T-DNA insertions of various P1/HC-Pro plants. The diagrammatic chromosomes were labeled with the T-DNA insertion positions and critical RNA silencing component genes. Bar, 100,000 bp. **c** AGO1 levels in various P1/HC-Pro plants. RUBISCO (asterisk) and tubulin were used as loading controls. **d** Immunoprecipitation for evaluating the interactions of AGO1 with various HC-Pros. The asterisks indicate the heavy chain of IgG. Tubulin was used as a loading control

Our previous study generated three viral species of P1/HC-Pro plants, including *P1/HC-Pro*^*Tu*^, *P1/HC-Pro*^*Zy*^, *P1/HC-Pro*^*Te*^, and *P1/HC-Pro*^*Tu−K*^ plants (Fig. 3a) [4, 8]. The *P1/HC-Pro*^*Tu*^, *P1/HC-Pro*^*Zy*^, and *P1/HC-Pro*^*Te*^ plants showed identical elliptical cotyledons (Fig. 3a, arrowheads). However, the cotyledons of *P1/HC-Pro*^*Tu−K*^ plants were round in shape similar to those of Col-0 (Fig. 3a). We applied NGS to obtain the genomic sequences from these P1/HC-Pro plants. We also developed T-DNA Finder software to identify the T-DNA insertion based on the genomic sequence profiles. Two T-DNAs were identified on chromosome 1: *P1/HC-Pro*^*Te*^ (at the 16,780,881^th^ nt) and *P1/HC-Pro*^*Tu*^ (at the 28,814,559^th^ nt) (Fig. 3b). In contrast, *P1/HC-Pro*^*Tu−K*^ was inserted on chromosome 3 (at the 21,554,188^th^ nt), whereas *P1/HC-Pro*^*Zy*^ was inserted on chromosome 4 (at the 10,075,981^th^ nt) (Fig. 3b). According to the TAIR database, several critical RNA silencing component genes, *e.g.*, AGOs, DCLs, RDRs, *HEN1*, *HYL1*, *SE*, and *ATG8a*, were also labeled on diagrammatic chromosomes with these T-DNA insertions, which is helpful for the generation of relative mutations in these P1/HC-Pro plants by crossing or gene editing (Fig. 3b).

We performed western blotting to evaluate the endogenous AGO1 levels in various transgenic plants, including the L1 line and *P1/HC-Pro*^*Tu*^ plants (Fig. 3c, panel i). Notably, the L1 line is a *β-glucuronidase* (*GUS*)-transgenic Arabidopsis plant that underwent sense-posttranscriptional gene silencing, and the T-DNA inserted on chromosome 3 (at the 2,350,388th nt) (Fig. 3b) [25]. The transgene of *P1/HC-Pro*^*Tu*^ was transferred into the L1 line to generate *P1/HC-Pro*^*Tu*^ plant [8]. The Col-0 and L1 lines showed identical endogenous AGO1 amounts, whereas *P1/HC-Pro*^*Tu*^ plants showed lower levels of AGO1 while HC-Pro was presented (Fig. 3c, panel i).

Moreover, Hu et al. demonstrated that various P1/HC-Pro plants have different AGO1 levels in vivo [4]. Indeed, *P1/HC-Pro*^*Tu*^ plants exhibited 0.5-fold AGO1 levels than Col-0 plants, whereas *P1/HC-Pro*^*Tu−K*^ plants had 0.7-fold AGO1 levels, suggesting that HC-Pro^Tu−K^ has a partial ability to trigger AGO1 degradation (Fig. 3c, panel ii). Notably, Hu et al. and Wei et al. demonstrated that low AGO1 protein levels are caused by autophagy instead of miR168-mediated RNA silencing. However, the AGO1 levels of the *P1/HC-Pro*^*Zy*^ and *P1/HC-Pro*^*Te*^ plants were 0.7- to 0.9-fold of those found in the wild-type plants, suggesting that various species of HC-Pros have different AGO1 degradation efficiency (Fig. 3c, panel ii) [4, 11]. Therefore, we aimed to understand whether endogenous AGO1 activity could be affected by VSR through in vitro RISC evaluation.

### Evaluation of the AGO1-HC-Pro interaction

A conserved WG pair, which localizes near the FRNK motif, is identified in 113 potyviruses [26]. Pollari et al. demonstrated that HC-Pro of potato virus A (HC-Pro^PVA^) physically interacts with AGO1 through transient expression with a cross-linked purification approach [26]. However, we performed AGO1-IP from various P1/HC-Pro plants with the α-AGO1 antibody. The IP results indicated that AGO1 could be detected in three P1/HC-Pro and Col-0 plants at the input and AGO1-IP samples (Fig. 3d). The immunoassay failed to detect HC-Pro in AGO1-IP samples from three P1/HC-Pro plants, suggesting that HC-Pro and AGO1 might not have a direct interaction but might exist a bridge protein to mediate an indirect interaction between HC-Pro and AGO1 (Fig. 3d).

### HC-Pro-mediated RISC inhibition assay

**Fig. 4:**
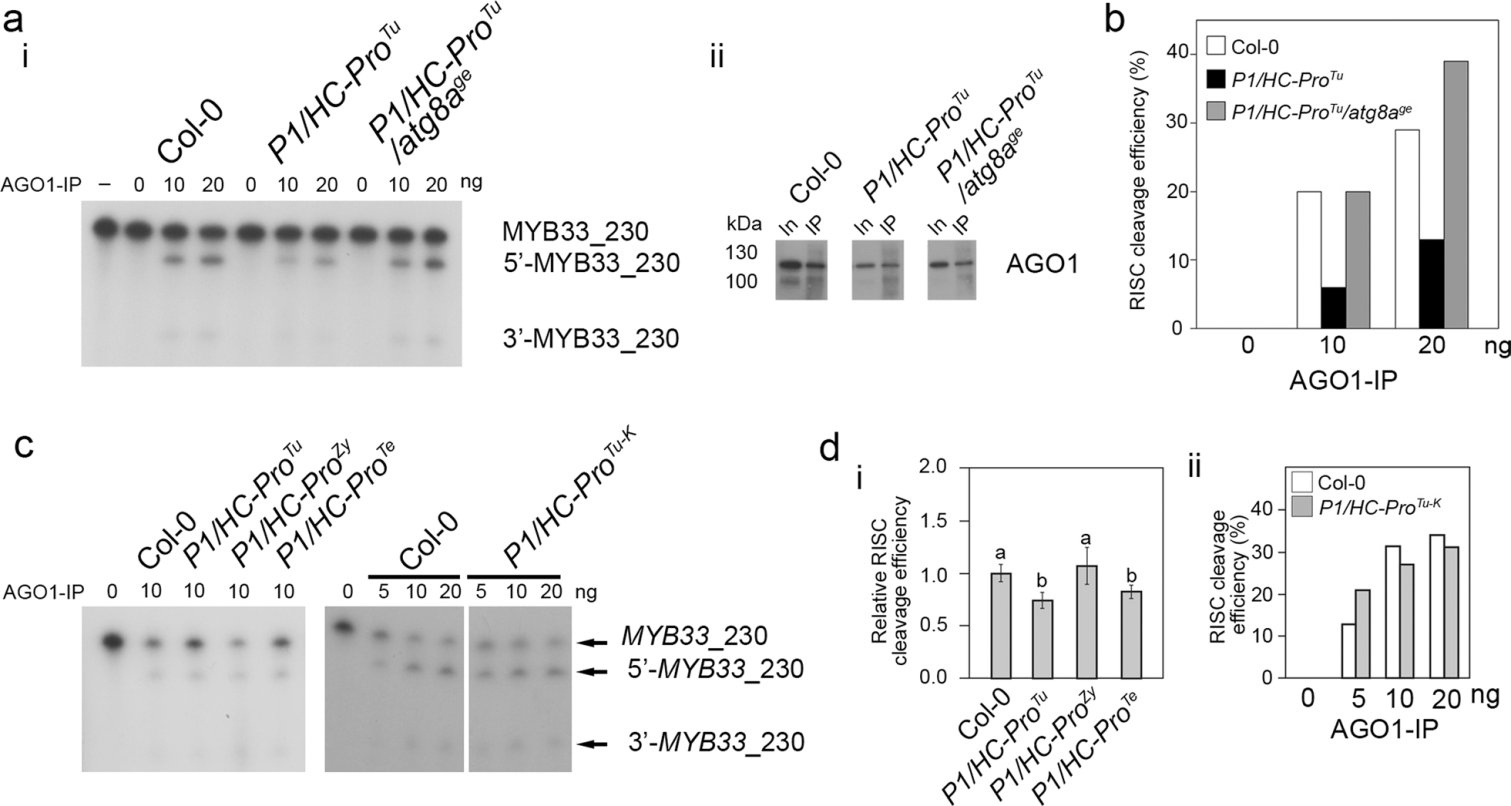
Evaluation of various HC-Pros in terms of their efficiency in inhibiting RISC activity in vitro. **a** In vitro RISC assay (i) and evaluation of the AGO1-IP efficiency (ii) for *P1/HC-Pro*^*Tu*^ and *P1/HC-Pro*^*Tu*^*/atg8a*^*ge*^ plants. In: input; IP: immunoprecipitation; FT: flow through. **b** Comparison of the in vitro RISC efficiency among the Col-0, *P1/HC-Pro*^*Tu*^, and *P1/HC-Pro*^*Tu*^*/atg8a*^*ge*^ plants. **c** Represented in vitro RISC assay with the *P1/HC-Pro*^*Tu*^, *P1/HC-Pro*^*Zy*^, *P1/HC-Pro*^*Te*^, and *P1/HC-Pro*^*Tu−K*^ plants from 3 biological repeats. **d** Relative RISC cleavage efficiency of the *P1/HC-Pro*^*Tu*^, *P1/HC-Pro*^*Zy*^, and *P1/HC-Pro*^*Te*^ plants (i) and comparison of the RISC efficiency between Col-0 and *P1/HC-Pro*^*Tu−K*^ plants (ii). The bars represent the standard deviations (*n* = 3). Means were compared by Tukey’s honestly significance test. Different letters above the bars indicate significant differences

We purified comparable amounts of AGO1-IP from the various transgenic plants for in vitro RISC assays to evaluate the status of AGO1 activity with *MYB33*-230 RNA substrates. The AGO1-IP from Col-0 seedlings exhibited 20% and 29% normalized cleavage efficiency in 10 ng and 20 ng AGO1-IP (Fig. 4a, panel i; and 4b). In contrast, although the AGO1-IP from the *P1/HC-Pro*^*Tu*^ plants still had RISC activity, the activity was reduced to 6% and 13% in 10 ng and 20 ng AGO1-IP, respectively, suggesting interference with RISC regulation (Fig. 4a, panel i; and 4b). Surprisingly, the normalized in vitro RISC activity from *P1/HC-Pro*^*Tu*^*/atg8a*^*ge*^ plants returned to 20% and 39% efficiency with 10 and 20 ng AGO1-IP, respectively, suggesting the restoration of RISC regulation (Fig. 4a, panel i; and 4b). Notably, the AGO1-IP from Col-0, *P1/HC-Pro*^*Tu*^, and *P1/HC-Pro*^*Tu*^*/atg8a*^*ge*^ plants were comparable (Fig. 4a, panel ii).

To evaluate other HC-Pros inhibit RISC activity, the *P1/HC-Pro*^*Zy*^ and *P1/HC-Pro*^*Te*^ plants were used to perform their RISC activity to compare with *P1/HC-Pro*^*Tu*^ and Col-0 plants (Fig. 4c). The relative RISC cleavage efficiency of *P1/HC-Pro*^*Zy*^ plants showed no difference from that of Col-0 but differed from that of *P1/HC-Pro*^*Tu*^ and *P1/HC-Pro*^*Te*^ plants, suggesting various HC-Pro of potyviruses have different RISC cleavage inhibition abilities. (Fig. 4c and 4d, panel i). Notably, the *P1/HC-Pro*^*Tu−K*^ sample did not inhibit RISC activity, suggesting FKNK mutation lost HC-Pro^Tu^-specific RISC inhibition ability (Fig. 4c and 4d, panel ii).

### TuMV-infected Arabidopsis triggers endogenous AGO1 degradation

**Fig. 5:**
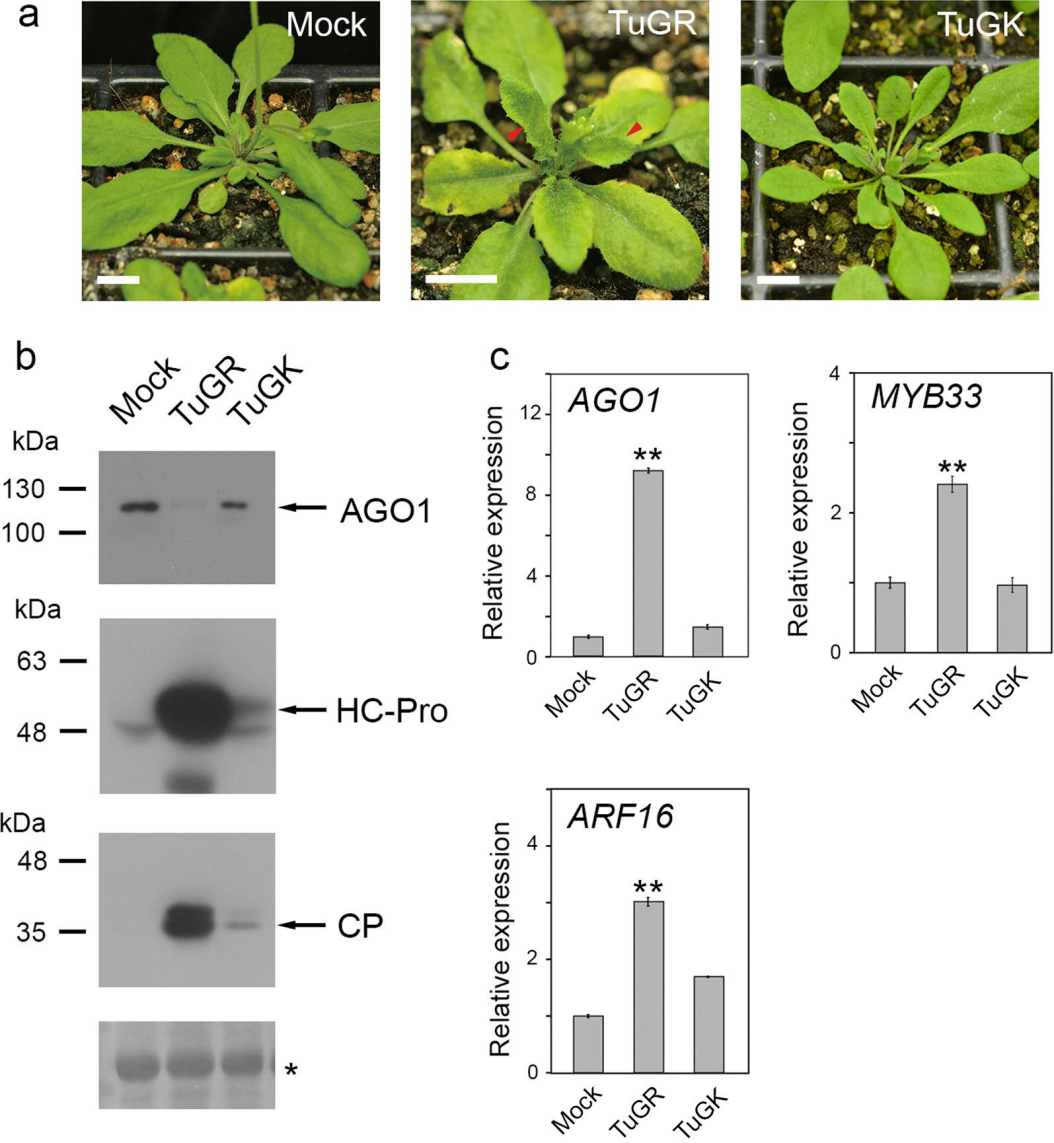
The AGO1 levels and evaluation of miRNA-mediated target cleavage in TuMV-infected plants. **a** Symptoms of TuGR- and TuGK-infected Col-0 plants at 12 dpi. Bar, 1 cm. The red arrowheads indicate the curing and serrated systemic leaves after TuGR infection. **b** Endogenous AGO1 and HC-Pro levels in mock, TuGR-, and TuGK-infected plants. The CP levels were used to confirm TuMV infection. RUBISCO (asterisk) was used as loading controls. **c** miRNA target gene expression of *AGO1* (miR168), *ARF16* (miR160), and *MYB33* (miR159) in mock, TuGR-, and TuGK-infected plants. The bars represent the standard errors (*n* = 3). The statistical significance was assessed based on Student’s *t* test. ** indicates *P* values < 0.01

We subsequently evaluated the endogenous AGO1 in TuMV-infected Col-0 plants (Fig. 5a). TuGR infection caused symptoms with mosaic and up-curling systemic leaves, whereas TuGK-infected plants showed symptomless (Fig. 5a). Similar to *P1/HC-Pro*^*Tu*^ plants, TuGR-infected Col-0 plants showed lower amounts of endogenous AGO1 than mock or TuGK-infected Col-0 plants (Fig. 5b). High abundances of HC-Pro and CP were detected in TuGR-infected plants, whereas lower amounts of HC-Pro and CP were detected in TuGK-infected Col-0 plants, which demonstrated uneven HC-Pro^Tu^ and HC-Pro^Tu−K^ amounts in infected tissues. Similarly, miRNA regulation suppression was also observed in TuGR-infected Col-0 plants. More than twofold higher levels of *AGO1* (miR168), *ARF16* (miR160), and *MYB33* (miR159) were also detected in TuGR-infected Col-0 plants (Fig. 5c). In contrast, TuGK infection induces *ARF16* accumulation to approximately 1.5-fold levels, whereas the RNA levels for *AGO1* and *MYB33* were similar to those obtained with the mock plants (Fig. 5c). These data indicated that miRNA regulation in TuGR-infected plants was also suppressed.

### TuMV infection inhibits the RISC activity

**Fig. 6:**
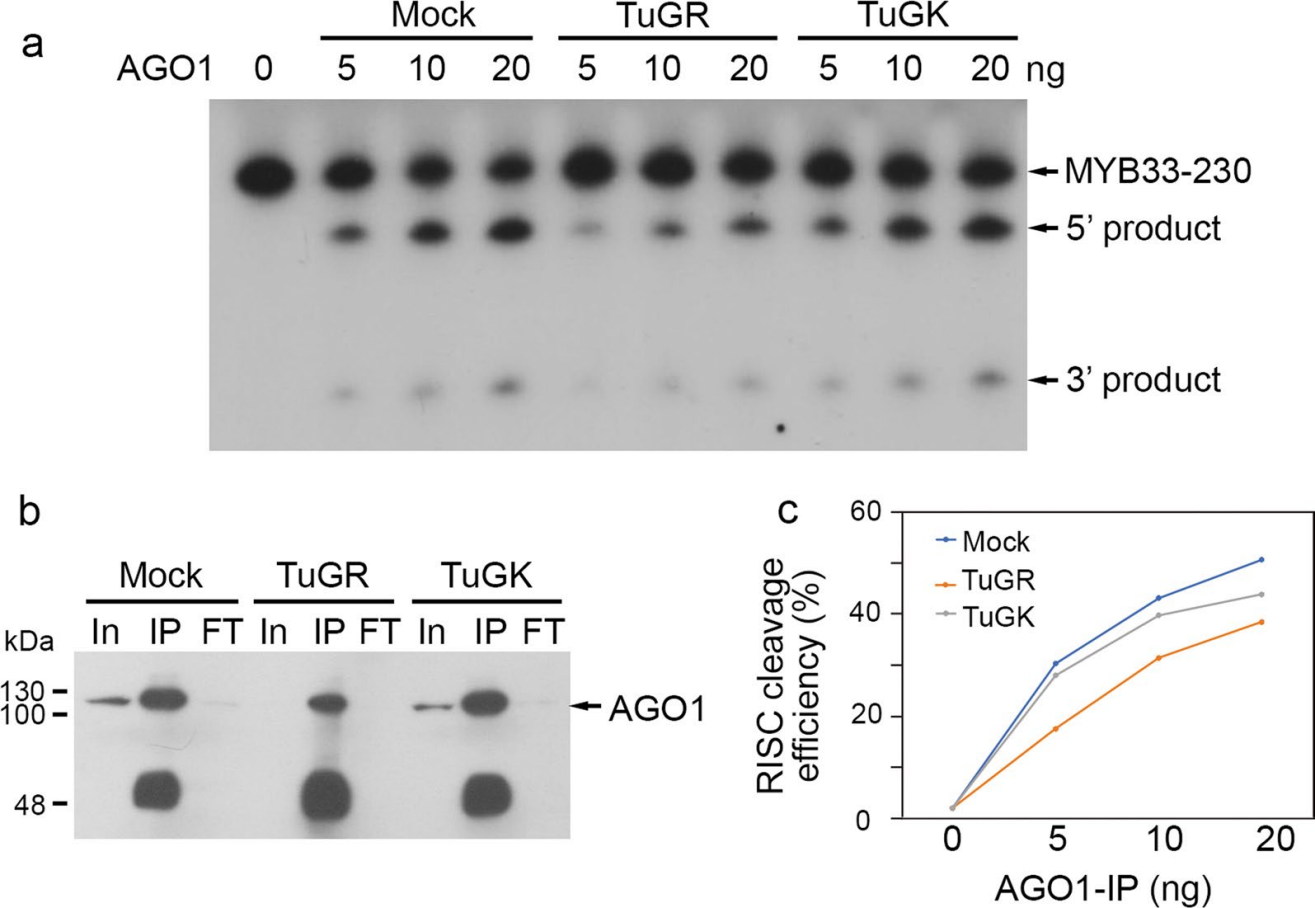
Evaluation of the in vitro RISC cleavage efficiency using TuMV-infected plants. **a** *MYB33*-230 RNA substrates were cleaved by AGO1-IP from mock, TuGR-, and TuGK-infected Col-0 plants. The numbers indicate the amounts of AGO1-IP that were used for cleavage. **b** The AGO1-IP amounts from mock, TuGR-, and TuGK-infected Col-0 plants were evaluated by western blotting with α AGO1 IgG to normalize the RISC cleavage efficiency. In: input; IP: immunoprecipitation; FT: flow through. **c** Normalized RISC cleavage efficiency of AGO1-IP from mock, TuGR-, and TuGK-infected Col-0 plants

We subsequently evaluated whether RISC activity might be affected during TuMV infection (Fig. 6a). We collected compatible amounts of AGO1-IP from mock, TuGR-, and TuGK-infected plants (Fig. 6b). The RISC assay demonstrated that the *MYB33*-230 substrate in TuGR-infected plants has lower cleavage efficiency compared with mock or TuGK-infected plants (Fig. 6c). As shown in Fig. 6c, 20 ng AGO1-IP from mock plants had 50.7% RISC activity, whereas 20 ng AGO1-IP from TuGR- and TuGK-infected plants had 38.5% and 43.9% RISC activity, respectively (Fig. 6a and c). These data demonstrated that severe TuMV infection inhibits RISC activity, which is consistent with the RISC results obtained in *P1/HC-Pro*^*Tu*^ plants.

### Discussion

Gain-of-function and loss-of-function genetic studies are fundamental approaches to understanding gene function. *Agrobacterium*-mediated transformation is popular for creating ectopic transgenic plants or T-DNA insertion mutants. However, a few studies have identified the T-DNA insertion position. Because NGS is more efficient and economical, identifying franking sequences of T-DNA insertion becomes achievable and with several benefits, including whether the insertion is on a gene body and which chromosome is inserted. In addition, transgenic plants with a transparent genetic background can introduce another mutation by crossing or CRISPR-Cas approaches. The T-DNA of *P1/HC-Pro*^*Tu*^ plants is inserted on chromosome 1 without breaking any gene body; thus, the *atg8a* mutant that localizes in chromosome 4 can be introduced into the transgenic plant to generate the *P1/HC-Pro*^*Tu*^*/atg8a*^*ge*^ plants [11, 16]. We also identified the other T-DNA insertions in these P1/HC-Pro plants. The insertion data with the locations of the RNA silencing component provide information for crossing strategy decisions. For instance, the T-DNA of the *P1/HC-Pro*^*Zy*^ plant is inserted on chromosome 4, which cannot introduce the *atg8a* mutant into this transgenic plant by crossing, and this deficiency might be overcome by gene editing. However, for introducing the other silencing mutants shown in Fig. 3b, the crossing approach can be applied to *P1/HC-Pro*^*Zy*^ plants. Moreover, we can generate double transgenic plants by crossing two homozygous VSR transgenic plants, *e.g.*, *P1/HC-Pro*^*Tu*^ plants × *P1/HC-Pro*^*Zy*^ plants, to study whether the synergistic effect would cause silencing suppression.

An in vitro RISC assay enables the direct analysis of AGO1 biological function. Baumberger et al. was the pioneering group that developed an in vitro RISC assay via IP of Flag-tagged AGO1 [17]. This system should be conducted in the *ago1-36* background to eliminate the impact of endogenous AGO1. Hence, establishing the Flag-AGO1 approach might be time-consuming for genetic screening and limit the utility of in vitro RISC assay to other mutants or transgenic plants when the simultaneous introduction of two elements, including the *Flag-AGO1* transgene and *ago1-36* locus, is needed. Moreover, the T-DNA insertion of Flag-AGO1 is unclear and might have a genetic linkage with the other mutants. T-DNA Finder can obtain the insertion information for Flag-AGO1/*ago1-36* plants. In contrast, the polyclonal α-AGO1 antibody targets the 240 aa N-terminus of AGO1 and specifically immunoprecipitates endogenous AGO1. We can rapidly take advantage of the in vitro RISC assay to verify the cleavage efficiency in any mutant and plant. *AGO1* has 56 different mutant alleles, and some alleles might lose certain functions [19]. For instance, *ago1-42* is mutated in the PAZ domain, which might affect the miRNA loading; whereas the a*go1-27* mutant might lose miRNA duplex binding [13, 24]. The AGO1-IP from these mutants can be used to perform small RNA profiling, RISC activity, and the RISC complex assembly. In contrast, viral-infected Arabidopsis can also be applied to investigate various VSRs in RISC inhibition efficiency. Virus infection of various *ago1* mutants can be used to investigate the interaction between VSRs and AGO1 through AGO1-IP with an α-AGO1 antibody.

Moreover, we demonstrated that the structure of the RNA substrate is critical for the in vitro RISC assay. The assay with the substrates *ARF10*-230 and *ARF10*-191 showed different cleavage efficiencies. We hypothesized that a targeting site length of the 3'-end might be essential. A 3'-end target site that is too long might form a complicated secondary structure, which may be difficult for AGO1 to access, as in the case of the *ARF10*-230 substrate. Thus, the RNA structure needs to be considered before determining the substrate sequence. We observed that miR159 and miR398 have more cleavage efficiency than miR164 and miR160. The amounts of miRNAs incorporated into AGO1 might cause a difference in the cleavage efficiency. The application of AGO1-IP with small RNA NGS can identify the AGO1-containing miRNA and siRNA profiles, which allows us to answer this question. Furthermore, AGO-IP from virus-infected plants can also obtain the viral siRNA profile, which can be applied in viral cleavage evaluation. AGO2 has been demonstrated to be involved in resistance to viral infection [27]. Therefore, we can apply the same approach to generate AGO2-specific antibodies for AGO2-IP and in vitro RISC assays in comparative studies.

Pollari et al. demonstrated that a WG pair of HC-Pro^PVA^ plays a role for AGO1 subcellular colocalization and interaction [26]. Wei et al. also demonstrated that HC-Pro^Tu^ colocalized with AGO1 but had no direct interaction, as revealed by Förster resonance energy transfer (FRET) analysis [11]. Indeed, the IP results from three P1/HC-Pro plants did not show that three HC-Pros directly interact with AGO1. However, Pollari et al. used a cross-linking approach to demonstrate the interaction of HC-Pro^PVA^ coupled with AGO1 [26]. It has been proposed that HC-Pro^Tu^ accelerates the aggregation of AGO1 and the host proteins to form RNA granules, which would recruit the colocalization of AGO1 and HC-Pro to cytoplasmic foci [28]. To summarize, we hypothesized that HC-Pro might interact with bridge host factors, which could also interact with AGO1, through the WG pair of HC-Pro, suggesting that HC-Pro and AGO1 might have an indirect interaction.

**Fig. 7:**
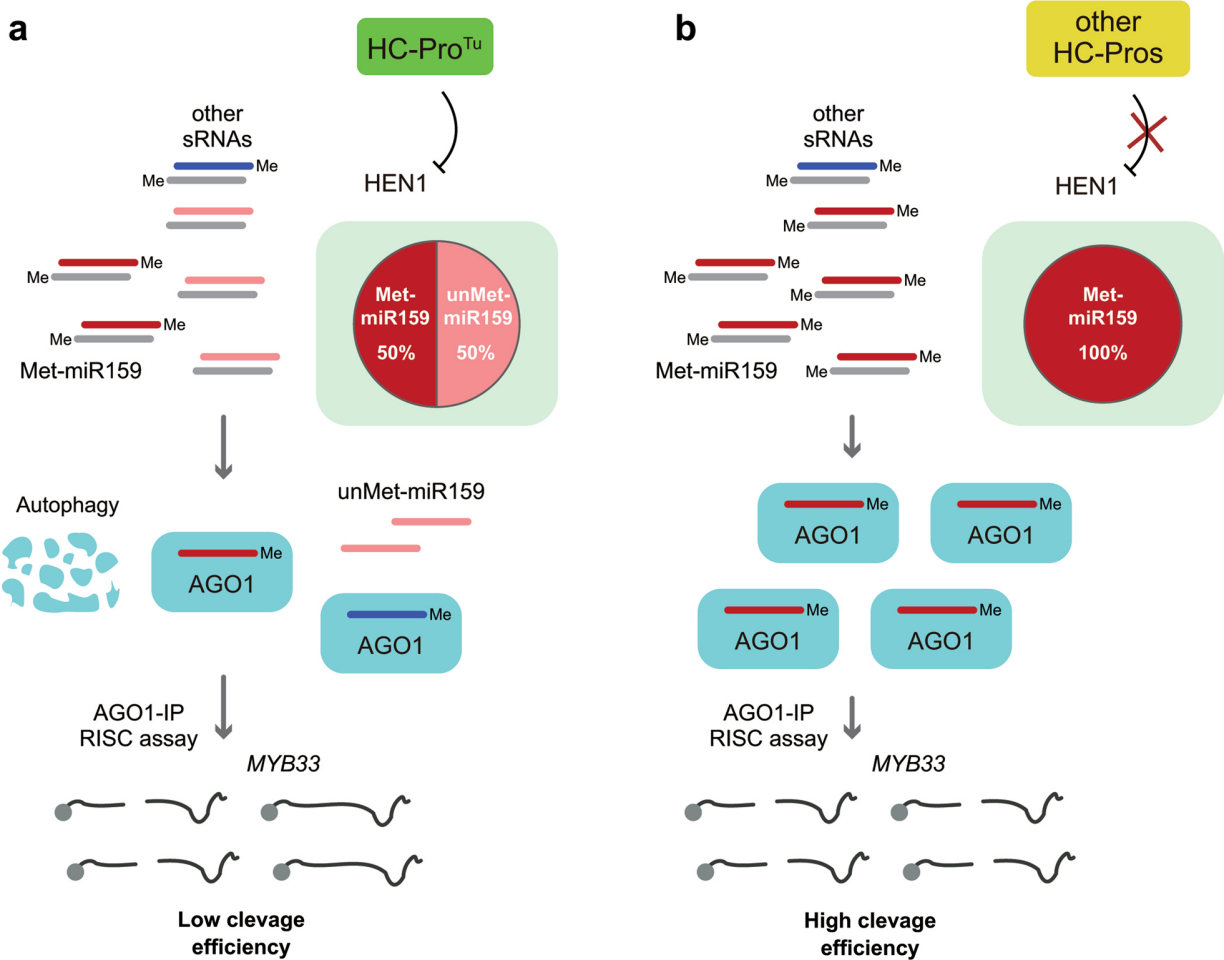
The working hypothesis for various HC-Pros in inhibiting the RISC activity. **a** The model of RISC inhibition by HC-Pro^Tu^. **b** The model to explain no effect on RISC inhibition by HC-Pro^Zy^ and HC-Pro^Te^. The dark red lines represent as mature miR159. The blue lines represent other small RNAs (sRNAs). The gray lines indicate the miRNA*. The dark red color indicates the methylated miRNAs. The pink color indicates the unmethylated miRNAs. Me, the methylated miRNA

Because comparable AGO1-IP amounts from three P1/HC-Pro plants, the more attenuated RISC activity of the P1/HC-Pro^Tu^ sample might not cause by decreased AGO1 levels. Wei et al. demonstrated that HC-Pro^Tu^ specifically inhibits HEN1 activity, resulting in 50% unmethylated miRNAs that could not be loaded into AGO1 (Fig. 7a) [11]. However, HC-Pro^Zy^ and HC-Pro^Te^ did not inhibit HEN1, which might allow more methylated miRNAs to be loaded into AGO1 for target RNA cleavage (Fig. 7b). We hypothesized that reduced miRNA content, *e.g.*, 50% methylated miR159, in AGO1-IP of P1/HC-Pro^Tu^ plants resulted in lower RISC cleavage efficiency (Fig. 7a). Although *P1/HC-Pro*^*Tu*^*/atg8a*^*ge*^ plants still contain approximately 50% unmethylated miRNAs [11]; however, restoring AGO1 in the plants allows them to carry more methylated miRNAs to restore the RISC activity (Fig. 4a and b). Moreover, *P1/HC-Pro*^*Te*^ plants containing 100% methylated miRNAs showed less RISC activity, suggesting that multiple RISC interference mechanisms might exist in different potyviruses. In summary, the reduced AGO1 amount and less methylated miRNAs in AGO1-IP are the unique abilities of HC-Pro^Tu^ to inhibit RISC activity, which means that TuMV can inhibit RISC activity more significantly compared with Col-0 and ZYMV-infected plants. In contrast, TEV infection may inhibit RISC less significantly than TuMV (Fig. 7).

Shiboleth et al. demonstrated that the FINK motif mutation of HC-Pro^Zy^ still retained vigorous RNA silencing suppression, suggesting small RNA sequestering is not the only way for HC-Pro^Zy^ inhibition RNA silencing but might exist multiple suppression approaches [6]. Wei et al. also demonstrated that HC-Pro^Zy^ and HC-Pro^Te^ still can slightly trigger AGO1 degradation [11]. Indeed, HC-Pro^Tu^ did not have a small RNA binding ability but can enhance autophagic AGO1 degradation and RISC inhibition more than HC-Pro^Zy^ and HC-Pro^Te^ because of HEN1 inhibition [11]. Therefore, we proposed that conserved and diverse VSR functions exist simultaneously in different viral HC-Pros.

## Conclusion

In this study, we demonstrated that T-DNA insertion information could be used to create more plant materials for experimental variations. The endogenous AGO1-based in vitro RISC assay can easily be performed with these materials for RNA silencing studies. Furthermore, our data imply that various HC-Pros of potyviruses exhibit different RISC inhibition and AGO1 degradation abilities in gene silencing suppression.

## Methods

### Plant materials and virus inoculum

*Arabidopsis thaliana* Columbia ecotype (Col-0), *P1/HC-Pro*^*Tu*^, *P1/HC-Pro*^*Tu−K*^, *P1/HC-Pro*^*Zy*^, *P1/HC-Pro*^*Te*^, *P1/HC-Pro*^*Tu*^*/atg8a*^*ge*^* plants*, *ago1-27*, and *ago1-36* mutants were used in this study [7, 8, 11, 16, 24]. The *ago1-27* mutant is an Ala992Val mutation at the C-terminus of AGO1, which is a weak allele mutant [24]. The *ago1-36* mutant (salk_087076) is an AGO1 null mutant with a T-DNA insertion at the end of the PAZ domain [17]. Arabidopsis seeds were surface sterilized and vernalized at 4 °C for 1 day before being sown on Murashige and Skoog medium. The wild-type TuGR, and the mild strain TuGK were maintained on *Nicotiana benthamiana* as inocula. The 2.5-week-old Arabidopsis seedlings were mechanically inoculated with viral inoculum prepared from 0.5 g of infected *N. benthamiana* leaves using 2 mL of 0.01 M potassium phosphate buffer (pH 7.2). The symptoms or subsequent analysis were observed 12 days post-inoculation (dpi).

### Antibody production

To produce the recombinant protein, the pET28a-AGO1-N plasmid was cloned and transformed into the *E. coli* BL21 system. The recombinant proteins were expressed in 500 mL LB culture and then induced with 0.5 mM IPTG at 20 °C overnight. Cells were suspended in 50 mL Denature Buffer (8 M urea, 0.1 M Na_2_HPO_4_, 20 mM Tris) with pH 8.0. Recombinant AGO1-N protein was purified with a 1 mL HisTrap FF column (Cytiva) and fast protein liquid chromatography (FPLC) (AKTA purifier, GE Healthcare). The procedure was manipulated following the FPLC user manual. A sample loop (Cytiva) was used for sample binding. Denature buffer with reducing pH was used to wash the column and to elute the protein. Two mg of AGO1-N was used as antigen for rabbit immunization. A total of 10 mL of serum was applied to a HiTrap Protein A FF column (Cytiva) via FPLC. The column was washed with 20 mL wash buffer (0.5 × PBS, 0.15 M NaCl, pH 7.4). IgG was eluted with 0.1 M glycine, pH 2.5, and dialyzed in 1 × PBS buffer at 4 °C overnight.

For IgG of AGO1-N affinity purification, the HiTrap NHS-Activated HP column (Cytiva) was used in this study. Recombinant protein at a concentration of 2 mg/mL was dialyzed in coupling buffer (0.2 M NaHCO_3_, 0.5 M NaCl, 2 M urea, pH 8.3) at 4 °C overnight. The antigen protein was coupled to NHS-activated columns following the user manual. For affinity purification, the purified IgG was passed through the antigen-coupled column in cycles with a flow rate of 0.5 mL/min for 2 h on FPLC. The column was washed with 10 mL 20 mM Tris–HCl, pH 7.5, and washed with 20 mL of a solution containing 20 mM Tris, pH 7.5 and 0.5 M NaCl. The affinity IgG was eluted with 0.1 M glycine (pH 2.5). The eluted IgG was dialyzed in 1 × PBS buffer at 4 °C overnight. IgG was diluted with 1 × PBS, and the same volume of glycerol was added to obtain the 1 mg/mL IgG of working concentration.

### Identification of the T-DNA insertion in various P1/HC-Pro plants

The genomic DNA was resequenced by next-generation sequencing (NGS) with approximately 20 million paired-end read throughput (150 nt × 150 nt). The CLC Genomics Workbench was used to generate SAM files based on the alignment results of resequencing reads with Arabidopsis genome and binary vector sequences. The SAM file was further processed to identify chimeric reads (imperfectly matched reads) using T-DNA Finder (github.com/ckhuang-git/T-DNA.Finder). These chimeric reads contained a perfectly matched region and a soft-clipped sequence. The soft-clipped sequences might contain vector sequences, Arabidopsis genomic DNA (gDNA), homopolymers or miscellaneous sequences. Soft-clipped sequences shorter than 50 nt or containing more than 10% read-length homopolymers were removed by T-DNA Finder. Soft-clipped sequences were isolated from the chimeric reads, and T-DNA Finder automatically analyzed these sequences using BLASTn with the nt database. If a soft-clipped sequence belongs to Arabidopsis, it will be exported as a text file for further analysis, such as for investigating T-DNA-mediated chromosome translocation. If a soft-clipped sequence belongs to a binary vector, it will be selected by T-DNA Finder, and the alignment between the reference sequence and chimeric read sequences will be exported as a PostScript file.

### AGO1-IP and in vitro RISC assay

For AGO1-IP, 15 μL IgG and 30 μL protein A beads (Cytiva, 17,152,104,011,150) were mixed and preincubated for 30 min following the user’s manual. Plant tissues (0.5 g) were ground, and the tissue lysate was extracted with 1 mL AGO1-IP buffer (50 mM Tris–HCl pH 7.5, 150 mM NaCl, 5 mM MgCl_2_, 0.1% Nonidet P-40, and 10% glycerol, 1 × Protein inhibitor, 5 mM DTT). The tissue lysate was centrifuged at full speed, and the supernatant was mixed with IgG beads. The reaction was incubated at 4 °C for 2 h. The AGO1 precipitates were then washed four times with IP buffer and then with 1 × PBS buffer. The amount of AGO1 in the IP product was quantified based on the standard recombinant AGO1-N protein by western blotting with the α-AGO1 antibody. The IP products were further used for small RNA extraction, western blotting, or in vitro RISC assay.

cDNA fragments, including *MYB33*-230, *MYB33*^mSeed^, *MYB33*^mCenter^, *CSD2*, *NAC1*-230, *ARF16*-230, *ARF10*-230, and *ARF10*-191, containing miRNA target sites were amplified with the primer sets detailed in Additional file 1: Table S1 and cloned into a pGEM-T easy vector (Promega). The DNA template for in vitro transcription was amplified for transcription. RNA was synthesized using the MEGAscript T7 Transcription Kit (Invitrogen) with the DNA template and the addition of 1 μL fresh [α-P^32^] UTP (PerkinElmer). Unincorporated UTP was removed by G50 resin (Cytiva). The RNA transcript was separated on a 6% denaturing PAGE gel in 1 × TBE buffer [29:1 acrylamide/bis (Bio-Rad), 8 M urea], and the PAGE gel was exposed to chemiluminescence film (Cytiva) to visualize the RNA. The band with a strong signal was excised and mashed in 300 μL extraction buffer (250 mM NaOAC, 1 mM EDTA) and passed through centrifuge tube filters (Costar). The supernatant was further cleaned up using the TRIzol method. RNA was diluted with 5 μL water to obtain a radioactive strength of 300 to 500 CPM. The original data of RISC assay are provided in Additional file 2, and these results have been showed in Figs. 2, 4, and 6, respectively.

The IP reaction for RISC activity was performed with 15 μL 1 mg/mL IgG, 30 μL beads, 500 μL lysate and ultimately resuspended in 50 μL 1 × PBS. For the slicer assay, 5 μL ^32^P-labeled substrate RNA (60 CPM/ μL) in 25 μL 2 × cleavage buffer [2 × PBS, 266.6 mM KCl, 26.6 mM MgCl_2_, 33.4 mM DTT, 6.6 mM ATP, 1.4 mM GTP, 0.25% RiboLock (Thermo Scientific)] incubated with AGO1-IP beads that contain 5, 10, or 20 ng AGO1, which quantified with standard concentrations of recombinant AGO1, at 25 °C for 1 h under rotation. The RNA fragment was purified by the TRIzol method. The RNA was solubilized in 10 μL water with 10 μL RNA Gel Loading Dye (Invitrogen) and denatured at 85 °C for 2 min. The RNA was separated on 6% denaturing PAGE gel and exposed to chemiluminescence film for 5 days to obtain the image.

The signals for full-length substrate, 5’- and 3'-cleaved fragments were detected using ImageJ. For calculation of the RISC cleavage efficiency, the product signal (5'-cleaved fragment + 3'-cleaved fragment) was divided by the total signal (full-length substrate + 5'-cleaved fragment + 3'-cleaved fragment), and the percentages were calculated. Each cleavage efficiency was normalized to the corresponding AGO1-IP signal, which was detected using ImageJ.

### Immunoblotting

For the detection of AGO1 protein in tissue lysates, 20 μL supernatant was diluted with 20 μL 1 × PBS buffer, and the lysate was denatured in 40 μL 2 × protein sample buffer. For IP-AGO1 detection, 90 μL 1 × PBS buffer and 100 μL 2 × protein sample buffer were added to 10 μL IP beads. The protein samples were boiled at 100 °C for 10 min.

The protein samples were separated and analyzed by polyacrylamide-SDS-PAGE. After electrophoresis, the separated proteins were transferred to a PVDF membrane by electroblotting. Polyclonal AGO1 (20,000 × dilution), coat protein (CP; 20,000 × dilution), and three HC-Pro (10,000 × dilution) IgGs were produced in our lab and used as the primary antibody. Monoclonal β-tubulin antibody (10,000 × dilution; sigma, T5168) was used for control. The signal was visualized by enhanced chemiluminescence detection with a Western Lightning™ Pro kit (PerkinElmer). The original images of western blot are provided in Additional file 2, and these results have been showed in Figs. 1–6, respectively.

## Supplementary Information

**Additional file 1: Table S1.** The primer list for this study.

**Additional file 2.** Supplementary Figures.

## Data Availability

The resequencing datasets generated during the current study are available in the NCBI Short Read Archive under accession numbers SRR6747669 (*P1/HC-Pro*^*Tu*^ plant), SRR22061796 (*P1/HC-Pro*^*Tu–K*^ plant), SRR22061797 (*P1/HC-Pro*^*TE*^ plant), and SRR22061798 (*P1/HC-Pro*^*Zy*^ plant).

## Abbreviations

AGO1: ARGONAUTE 1
AGO2: ARGONAUTE 2
AGO4: ARGONAUTE 4
AGO10: ARGONAUTE 10
ARF16: AUXIN RESPONSE FACTOR 16
GFP: Green fluorescent protein
GUS: β-Glucuronidase
RISC: RNA-inducing silencing complex
TuMV: Turnip mosaic virus
ZYMV: Zucchini yellow mosaic virus
TEV: Tobacco etch virus
siRNA: Short-interfering RNA
miRNA: MicroRNA
HC-Pro^Zy^: ZYMV HC-Pro
HC-Pro^Tu^: TuMV HC-Pro
HC-Pro^Te^: TEV HC-Pro
DCLs: Dicer-like proteins
RDRs: RNA-dependent-RNA polymerases
HYL1: HYPONASTIC LEAVES1
SE: SERRATE
HEN1: HUA ENHANCE 1
VSRs: Viral suppressors of RNA silencing
FRET: Förster resonance energy transfer
