# Supplementary material for: Development of an assay system for the analysis of host RISC activity in the presence of a potyvirus RNA silencing suppressor, HC-Pro
Source: Virol J. 2023 Jan 17;20:10. doi: 10.1186/s12985-022-01956-2 (PMC9844029; doi:10.1186/s12985-022-01956-2)
Supplement: Supplementary file 1 — Additional file 1: Table S1. The primer list for this study. [file 12985_2022_1956_MOESM1_ESM.docx]

**Additional file 1: Table S1. The primer list for this study.**

| **For AGO1-N cloning** | |
| --- | --- |
| For-AGO1-N | 5'-TATGGCTAGCATGGTGAGAAAGAGAAGAAC-3' |
| Rev-AGO1-N | 5'-GGTGCTCGAGGTGAGAATCACGATAATTAT-3' |
| **For MYB33-230 and miRNA target site mutagenesis** | |
| For-T7-MYB33 | 5'-TAATACGACTCACTATAGGGATTTCCAAAACTTGCAGCTTCTCAG-3' |
| Rev-MYB33-230 | 5'-GGAGATCAGAGTGTGGAGGAG-3' |
| P-HindIII-MYB33-mSeed | 5'-AGTGAAGCTTTGGAGCTCCCTTCATTAGAATATTC-3' |
| P-HindIII-MYB33-mCenter | 5'-AGTGAAGCTTTGGAGCTCCCAACATTCCAATATTC-3' |
| **For other target gene cloning** | |
| P-T7-CSD2-miR398b-3p | 5'-TAATACGACTCACTATAGGGTTGTGAATGTTCGTATCACTGGTCTC-3' |
| M_CSD2_miR398b-3p | 5'-TCAGAGGAATCTGATTGTCCACTAT-3' |
| P-T7-SPL9-miR157a-5p | 5'-TAATACGACTCACTATAGGGTTGAGGCGGCCAGTGTCGTCACCGTC-3' |
| M-SPL9-miR157a-5p | 5'-GCTCGCCATGTATTGTTGTTGTTGT-3' |
| P-T7-NAC1 | 5'-TAATACGACTCACTATAGGGTACGGAAGGAGTTATATGTAGAGAC-3' |
| M-NAC1 | 5'-TCTTGAGTTCAGAGACTGAGTTGGT -3' |
| P-T7-ARF16 | 5'-TAATACGACTCACTATAGGGACTTCGTTTTCGCCACCGAGGAAAA |
| M-ARF16 | 5'-CTATTCAAGTAGTAATGGTGAAGAT-3' |
| P-T7-ARF10-miR160-230 | 5'-TAATACGACTCACTATAGGGTTCTCTCCGAGGAAGAAGATTAGGA-3' |
| P-T7-ARF10-miR160-191 | 5'-TAATACGACTCACTATAGGGTGAGTTTCCATTCCACGGTACTAAAT-3' |
| M-ARF10 | 5'-CTACTAAGATTGAGATCAGACAACA-3' |
| P-T7-TuMV5403 | 5'-TAATACGACTCACTATAGGTTATGTGTCGCGACATCCCAGAAAA-3' |
| M-TuMV5403 | 5'-CTGCTTTCTTCTCTCCTCAG-3' |
| P-T7-TuMV7302 | 5'-TAATACGACTCACTATAGGAGTGTGGAACGGCTCGTTGAAGGCA-3' |
| M-TuMV7302 | 5'-ATCAGCATCACAATACACCC-3' |
| P-T7-TuMV8119 | 5'-TAATACGACTCACTATAGGGAGATAATGCTGAGAAATTTGTACA-3' |
| M-TuMV8119 | 5'-CTCCGTTGACGAAGAATCTG-3' |
| P-T7-TuMV9221 | 5'-TAATACGACTCACTATAGGTGATGGACGGCGATGATCAGGTGGA-3' |
| M-TuMV9454 | 5'-CGTATTGGAGTTCTAGAAGT-3' |
| P-T7-TuMV-7830 | 5'-TAATACGACTCACTATAGGGCTCGTTGAAGGCAGAGTTACGAC-3' |
| M-TuMV-7830 | 5'-AACAAACGATCCCAACCACAA-3' |
| **For qPCR** | |
| P_MYB33_qPCR | 5'-GCATCGAGCTGGCTTGACC-3' |
| M_MYB33_qPCR | 5'-ACCCAACTCCTGAGGATGCC-3' |
| P_AGO1_qPCR | 5'-CTCAGGTGGTGGACGTGGTT-3' |
| M_AGO1_qPCR | 5'-TTGCTGTTGTGGTGGTTGCC-3' |
| P_ARF16_qPCR | 5'-GCCCGTTAAGCTCTGTTCTGG-3' |
| M_ARF16_qPCR | 5'-TGGAGGAGGAGGAGGAGGTG-3' |
