# Supplementary figures and images for: Development of an assay system for the analysis of host RISC activity in the presence of a potyvirus RNA silencing suppressor, HC-Pro
Source: Virol J. 2023 Jan 17;20:10. doi: 10.1186/s12985-022-01956-2 (PMC9844029; doi:10.1186/s12985-022-01956-2)

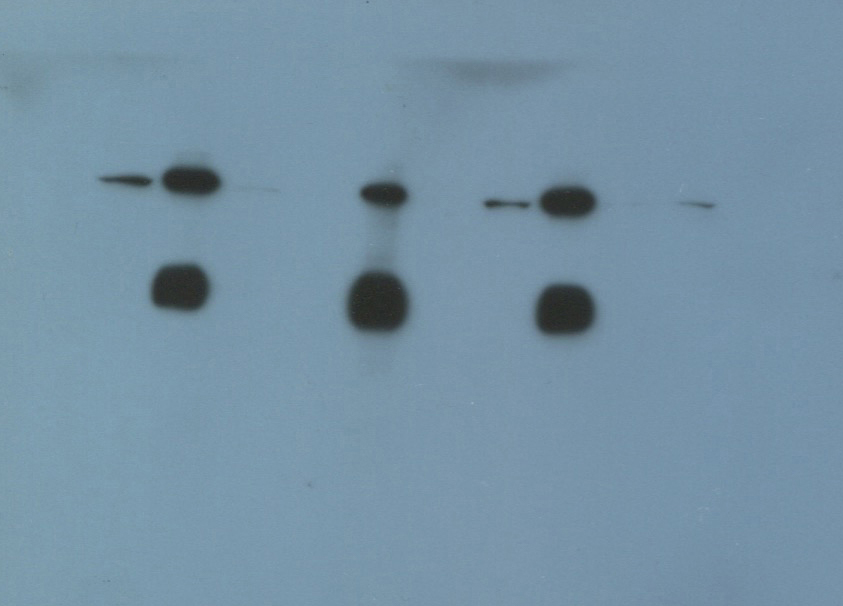

Supplement: Supplementary file 2 — Additional file 2. Supplementary Figures. [file 12985_2022_1956_MOESM2_ESM.zip › Additional file 2_Original images/Fig6b_AGO1-IP.jpg]

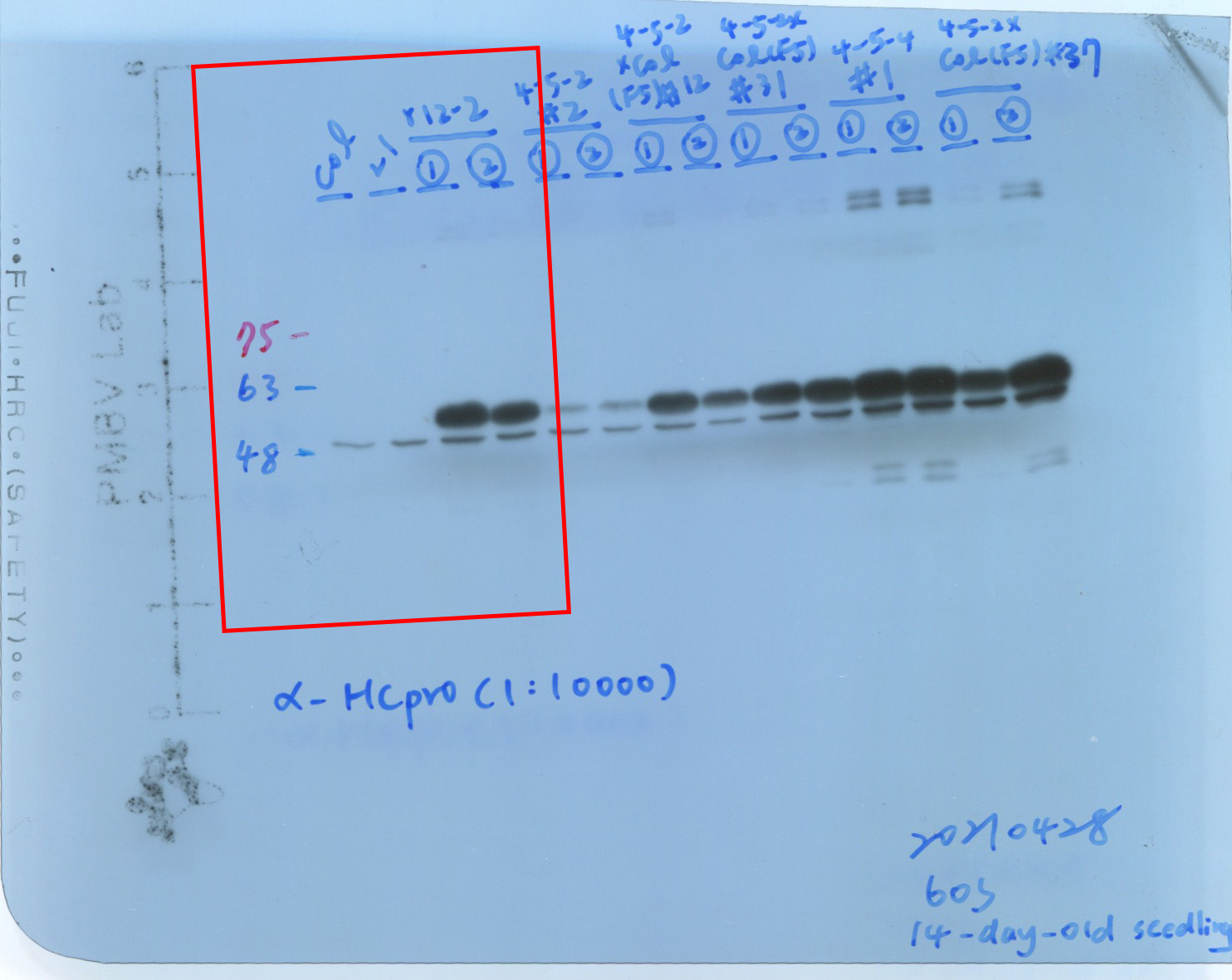

Supplement: Supplementary file 2 — Additional file 2. Supplementary Figures. [file 12985_2022_1956_MOESM2_ESM.zip › Additional file 2_Original images/Fig3c_HC-Pro Label.jpg]

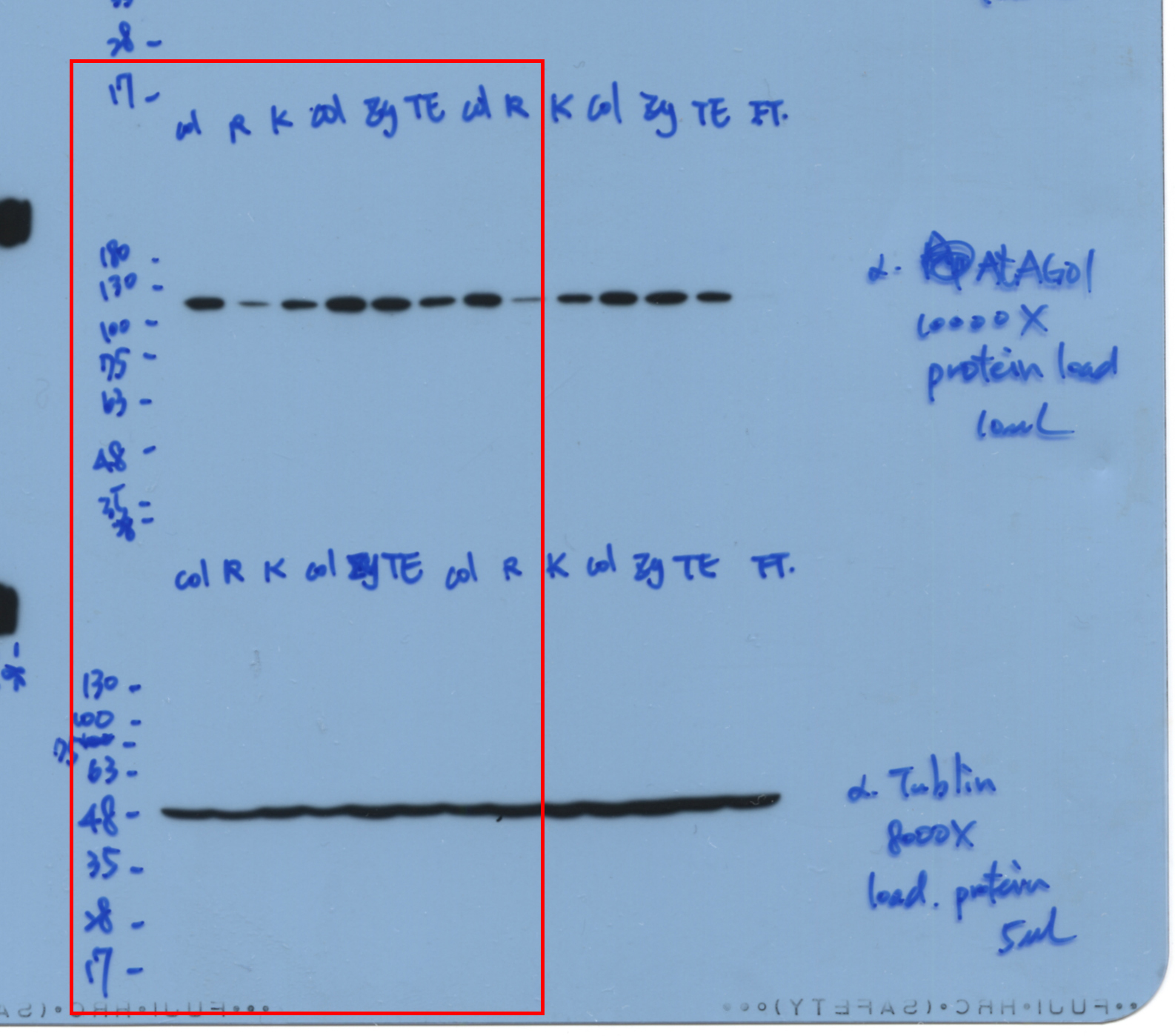

Supplement: Supplementary file 2 — Additional file 2. Supplementary Figures. [file 12985_2022_1956_MOESM2_ESM.zip › Additional file 2_Original images/Fig3cii_AGO1 Label.jpg]

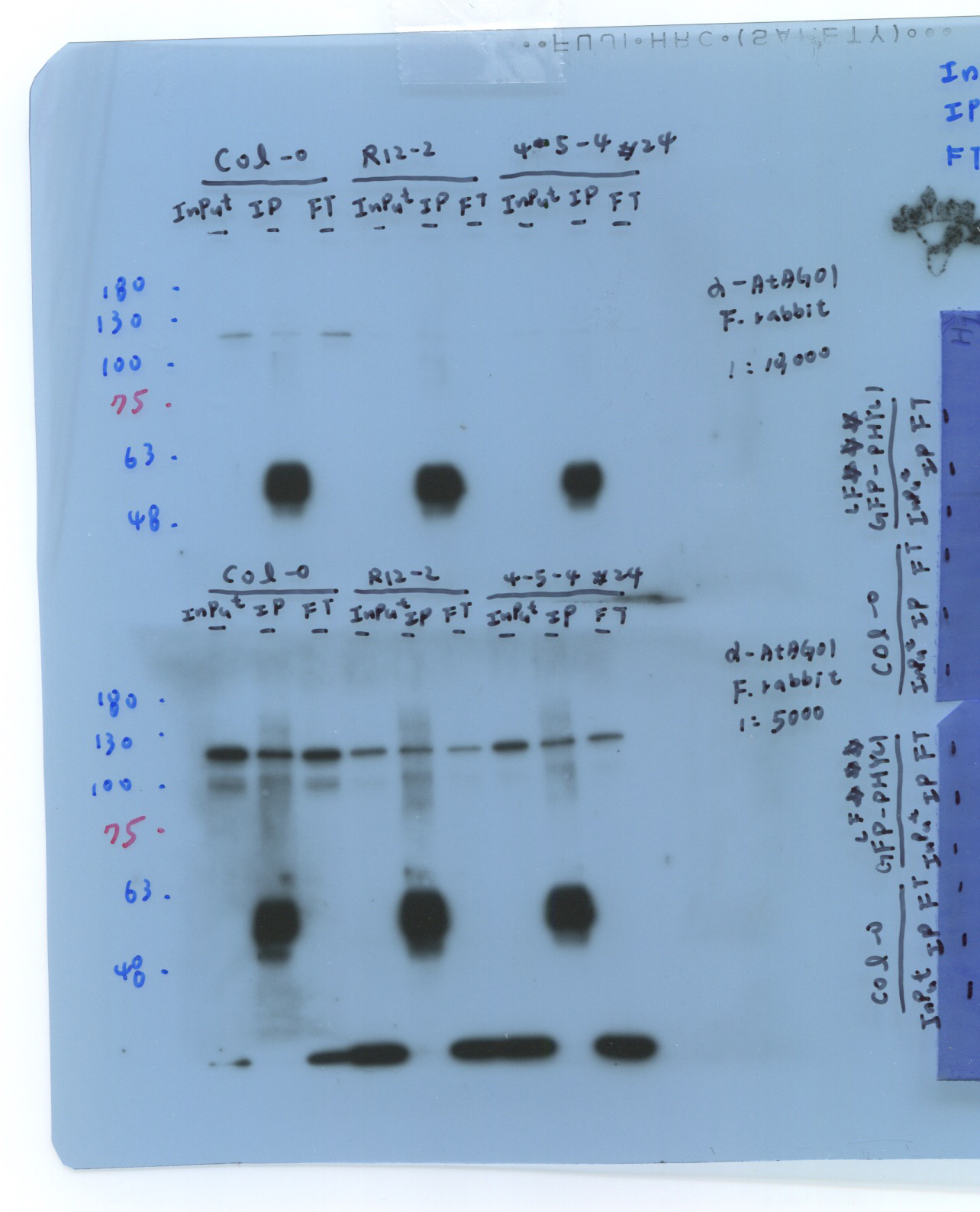

Supplement: Supplementary file 2 — Additional file 2. Supplementary Figures. [file 12985_2022_1956_MOESM2_ESM.zip › Additional file 2_Original images/Fig4aii.jpg]

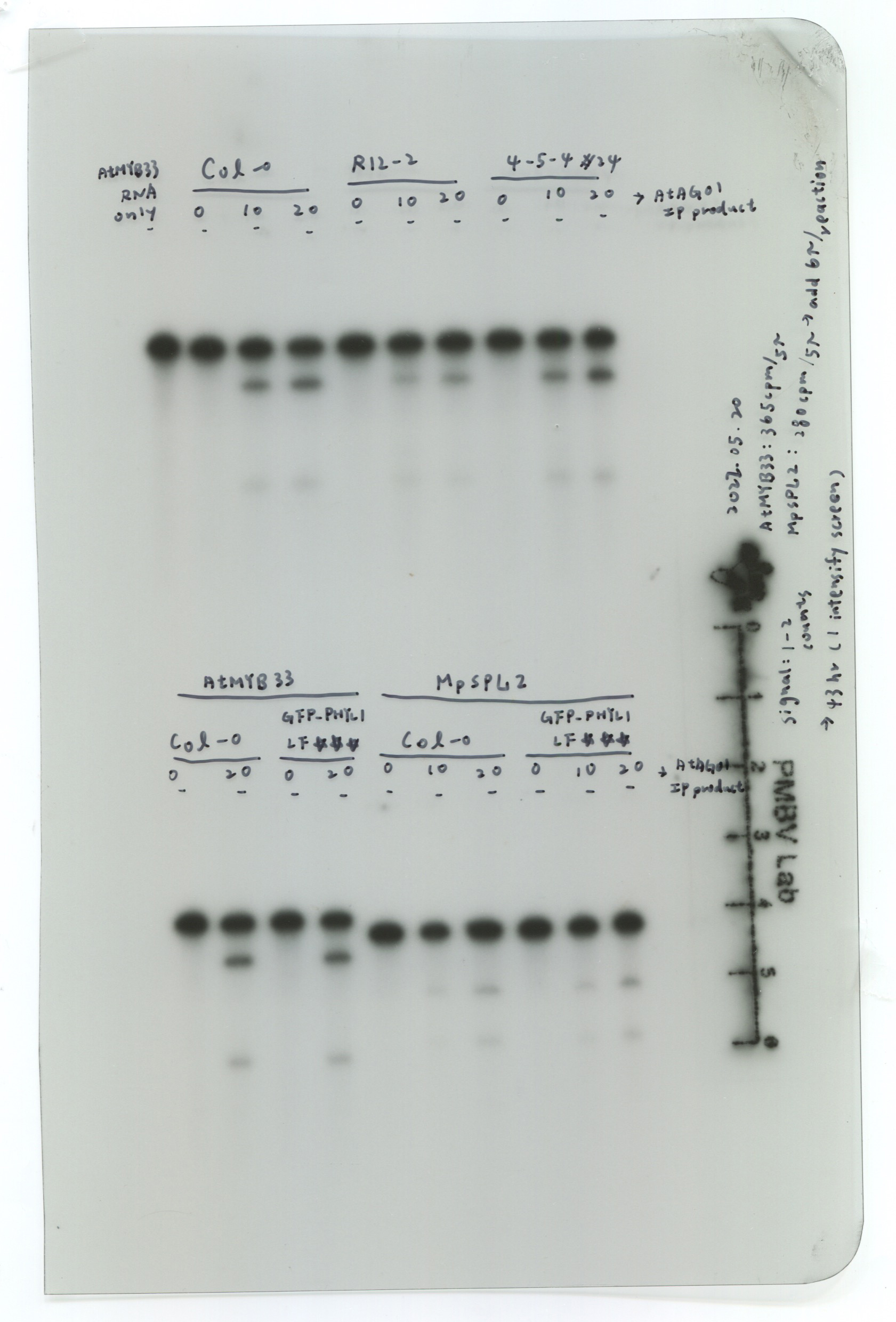

Supplement: Supplementary file 2 — Additional file 2. Supplementary Figures. [file 12985_2022_1956_MOESM2_ESM.zip › Additional file 2_Original images/Fig4ai.jpg]

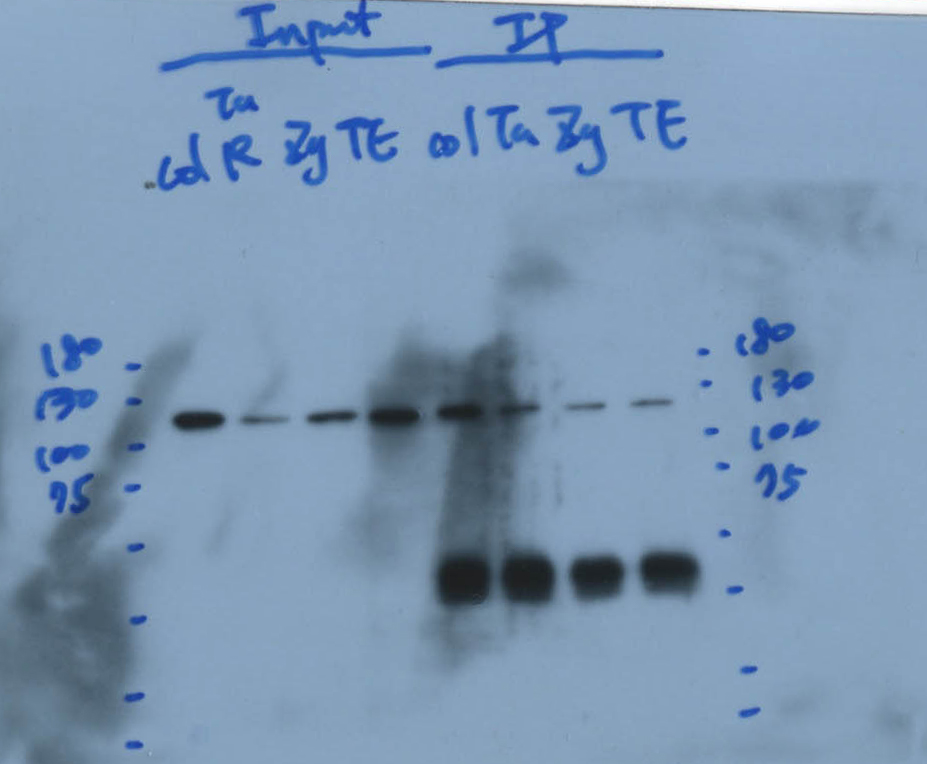

Supplement: Supplementary file 2 — Additional file 2. Supplementary Figures. [file 12985_2022_1956_MOESM2_ESM.zip › Additional file 2_Original images/Fig3d_AGO1.jpg]

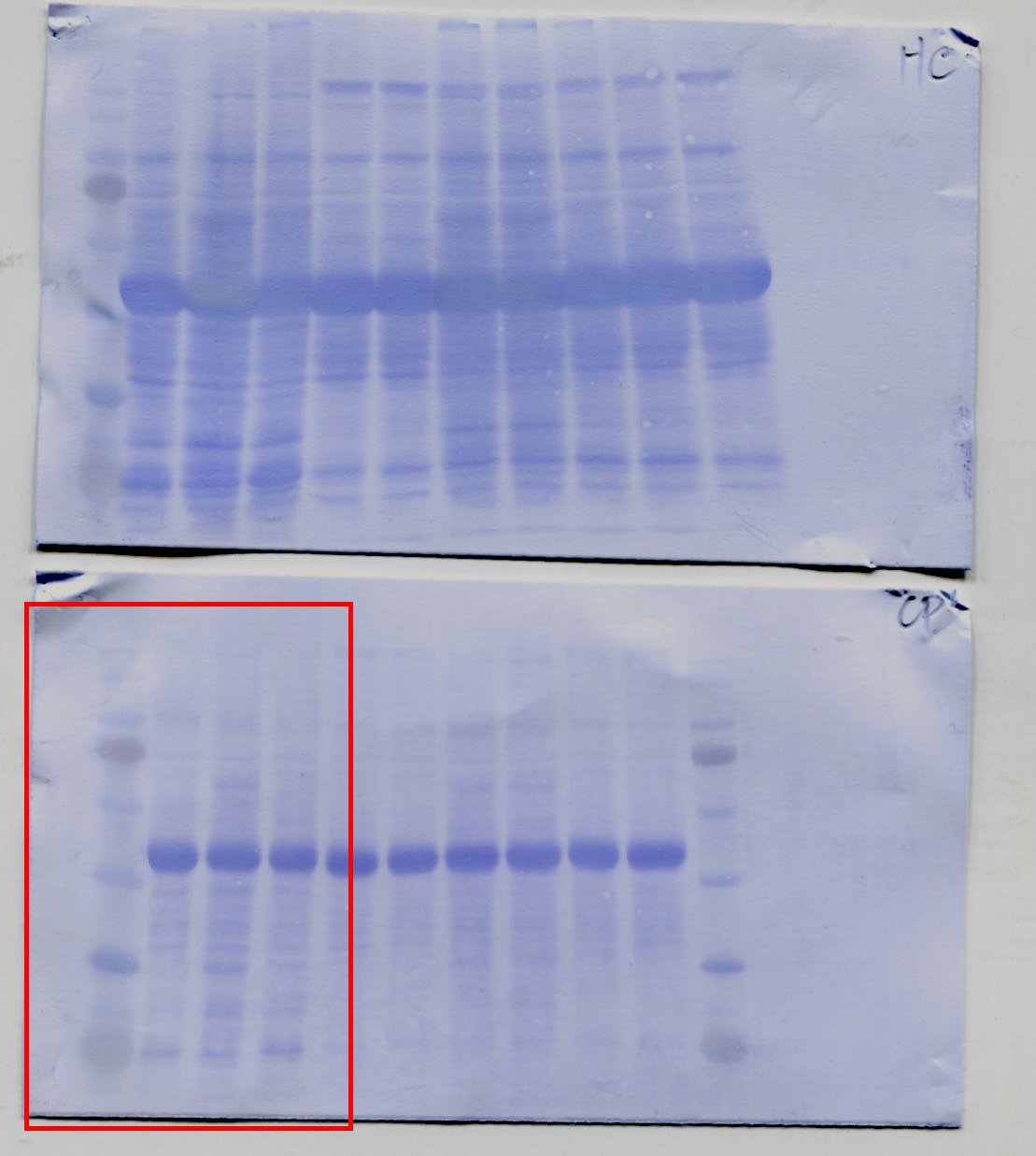

Supplement: Supplementary file 2 — Additional file 2. Supplementary Figures. [file 12985_2022_1956_MOESM2_ESM.zip › Additional file 2_Original images/Fig5b_RUBISCO Label.jpg]

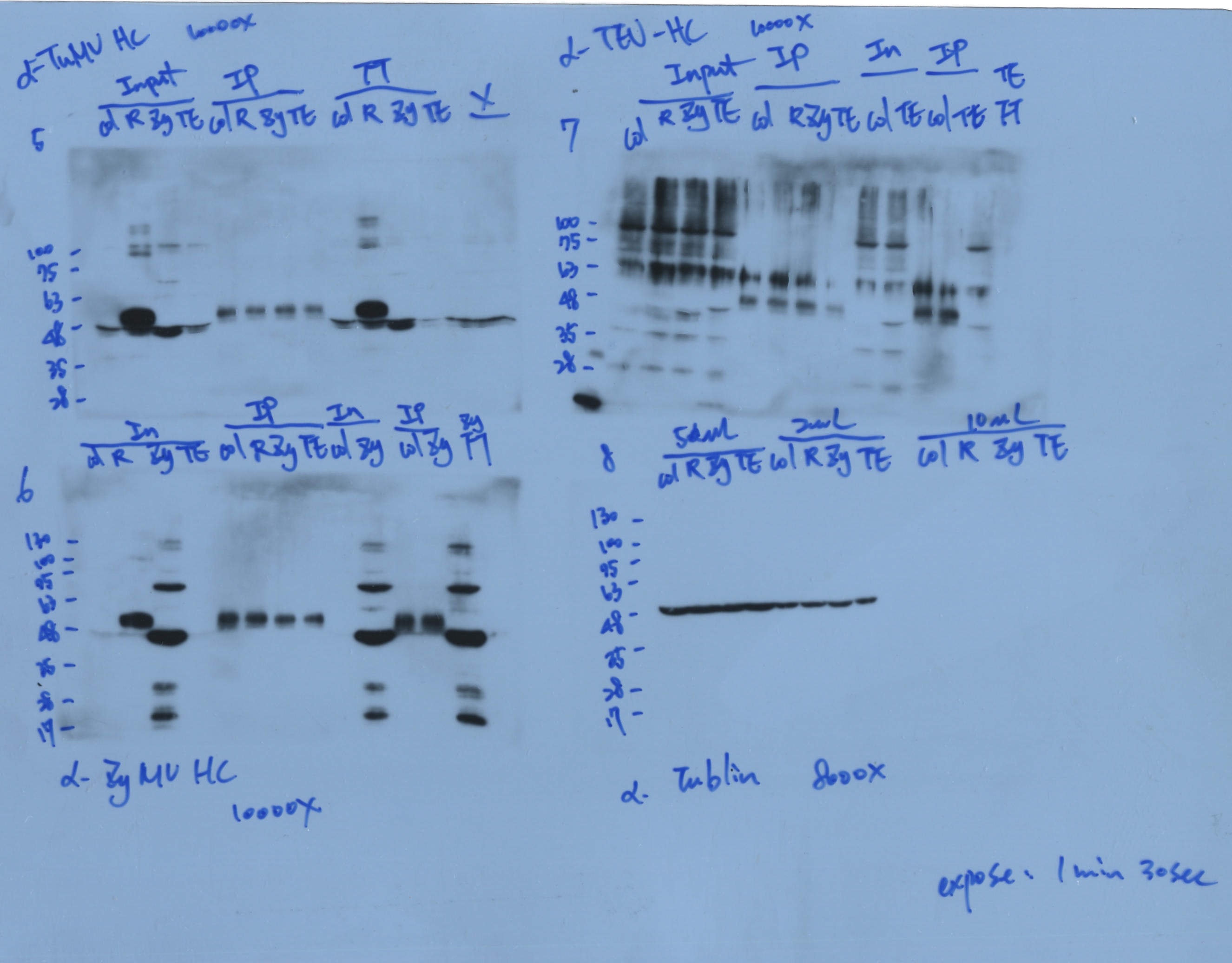

Supplement: Supplementary file 2 — Additional file 2. Supplementary Figures. [file 12985_2022_1956_MOESM2_ESM.zip › Additional file 2_Original images/Fig3d_HC_Tubulin.jpg]

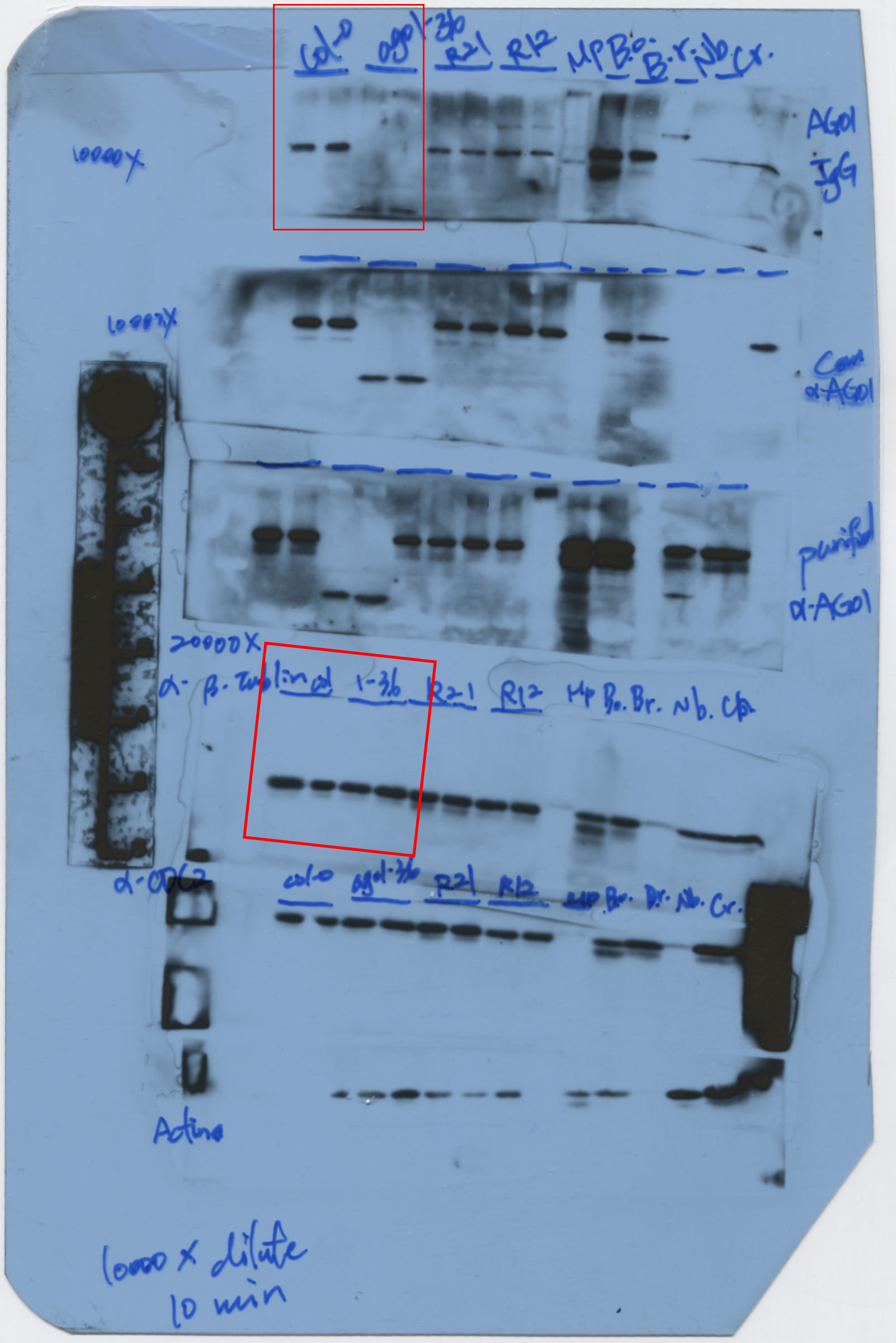

Supplement: Supplementary file 2 — Additional file 2. Supplementary Figures. [file 12985_2022_1956_MOESM2_ESM.zip › Additional file 2_Original images/Fig1b Label.jpg]

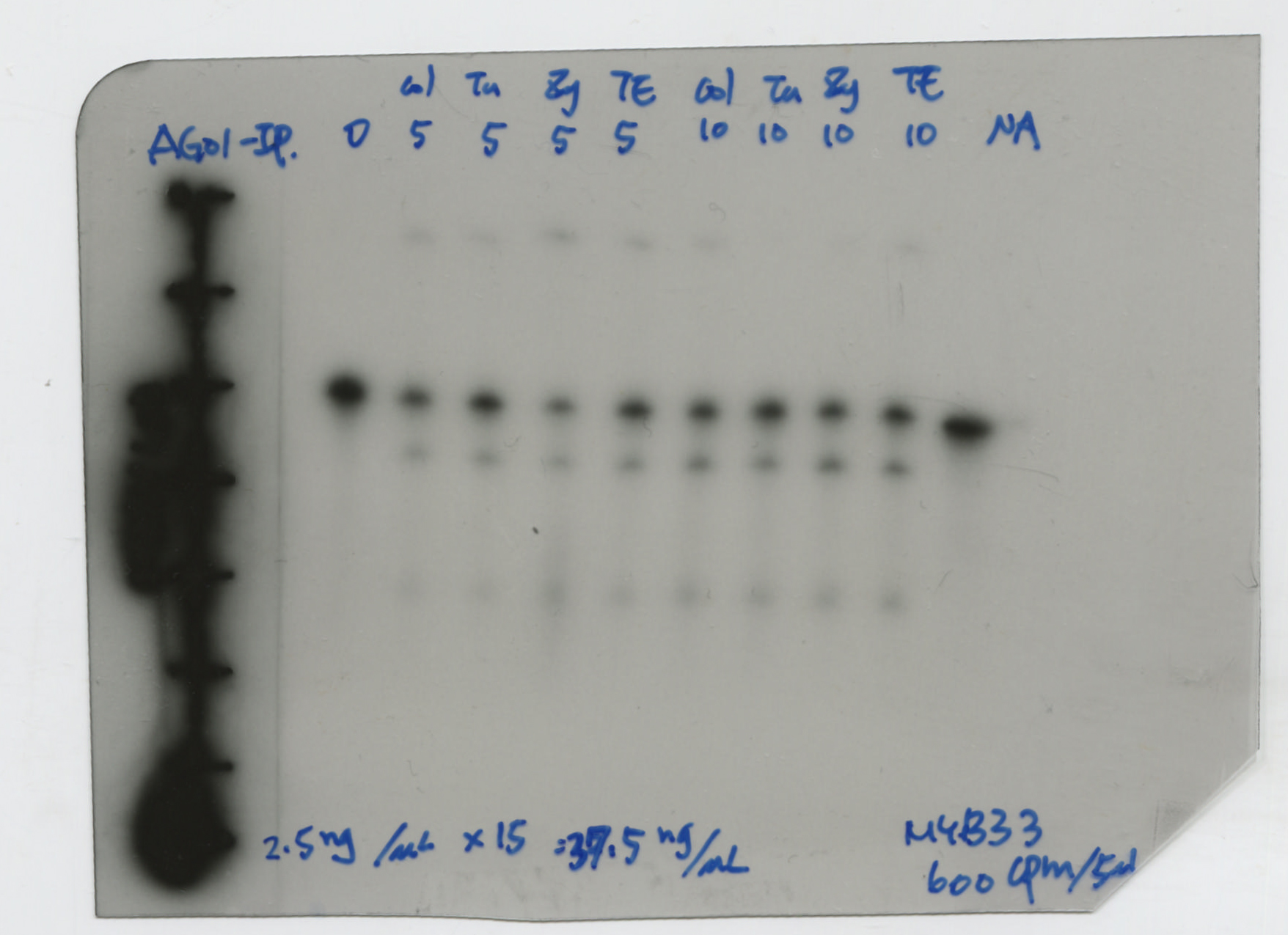

Supplement: Supplementary file 2 — Additional file 2. Supplementary Figures. [file 12985_2022_1956_MOESM2_ESM.zip › Additional file 2_Original images/Fig4c left.jpg]

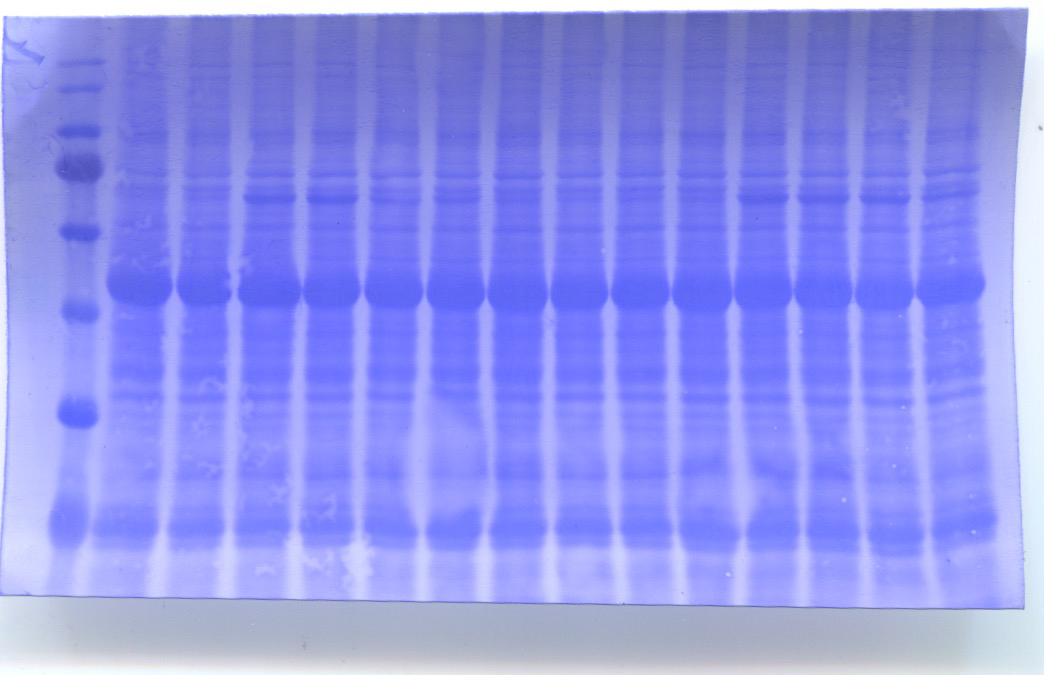

Supplement: Supplementary file 2 — Additional file 2. Supplementary Figures. [file 12985_2022_1956_MOESM2_ESM.zip › Additional file 2_Original images/Fig3c_RUBISCO.jpg]

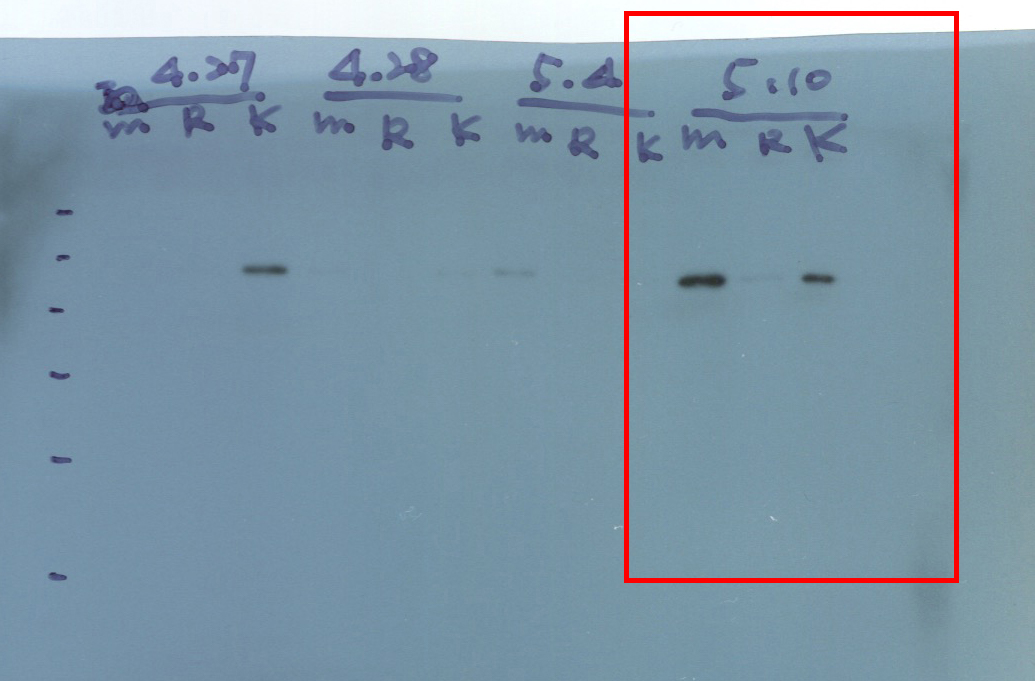

Supplement: Supplementary file 2 — Additional file 2. Supplementary Figures. [file 12985_2022_1956_MOESM2_ESM.zip › Additional file 2_Original images/Fig5b_AGO1.jpg]

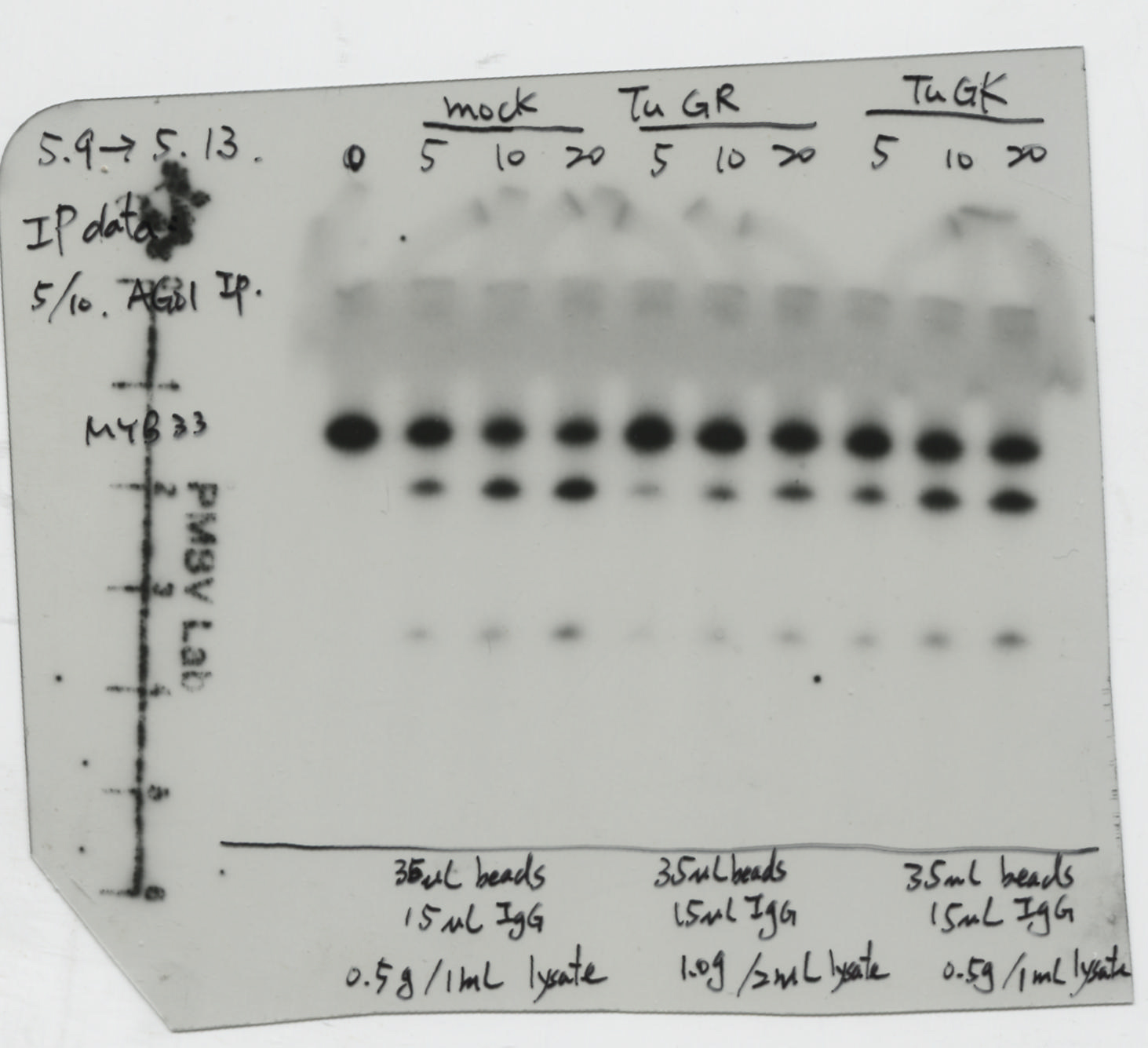

Supplement: Supplementary file 2 — Additional file 2. Supplementary Figures. [file 12985_2022_1956_MOESM2_ESM.zip › Additional file 2_Original images/Fig6a.jpg]

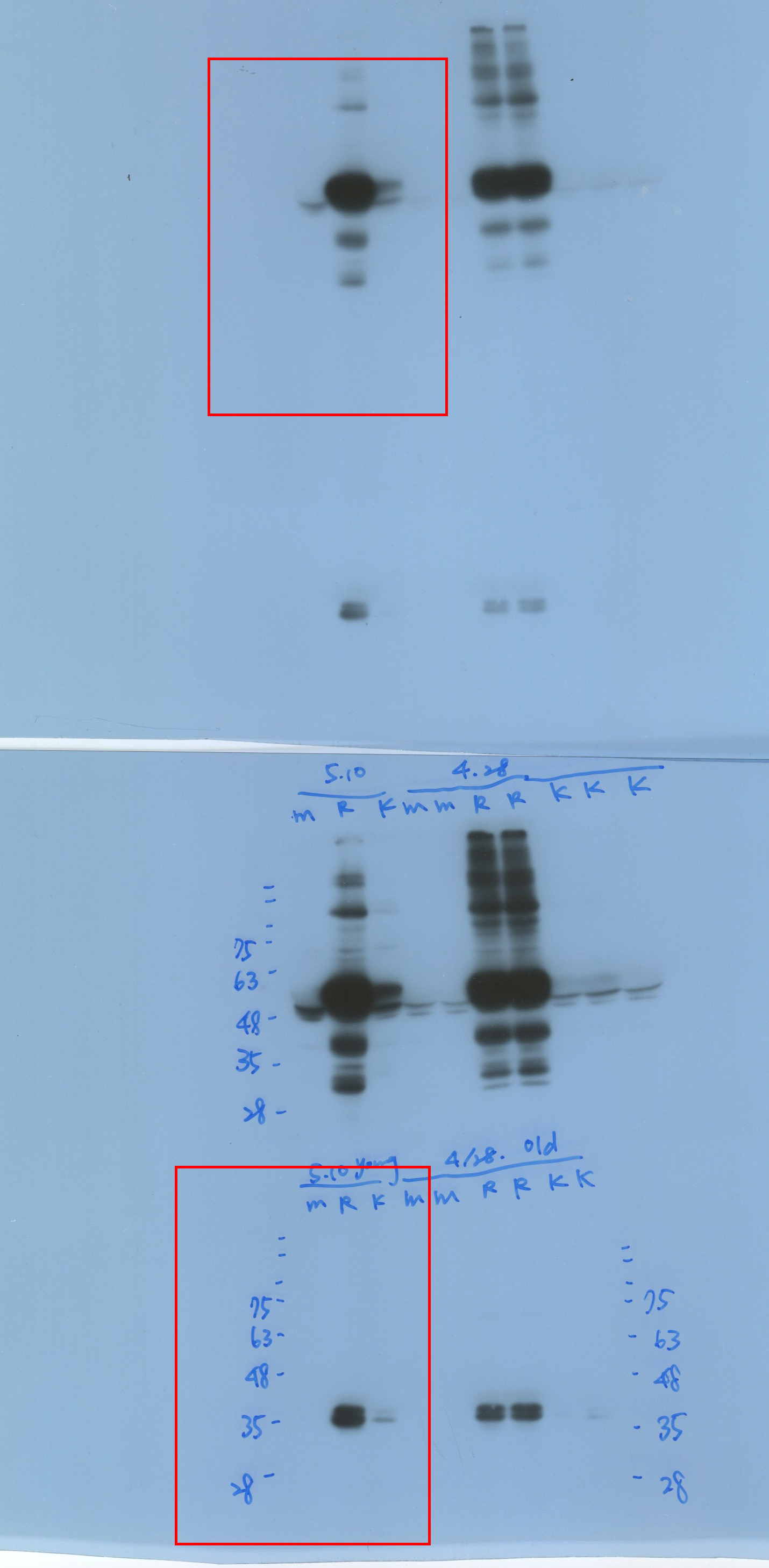

Supplement: Supplementary file 2 — Additional file 2. Supplementary Figures. [file 12985_2022_1956_MOESM2_ESM.zip › Additional file 2_Original images/Fig5b_HC-Pro_CP Label.jpg]

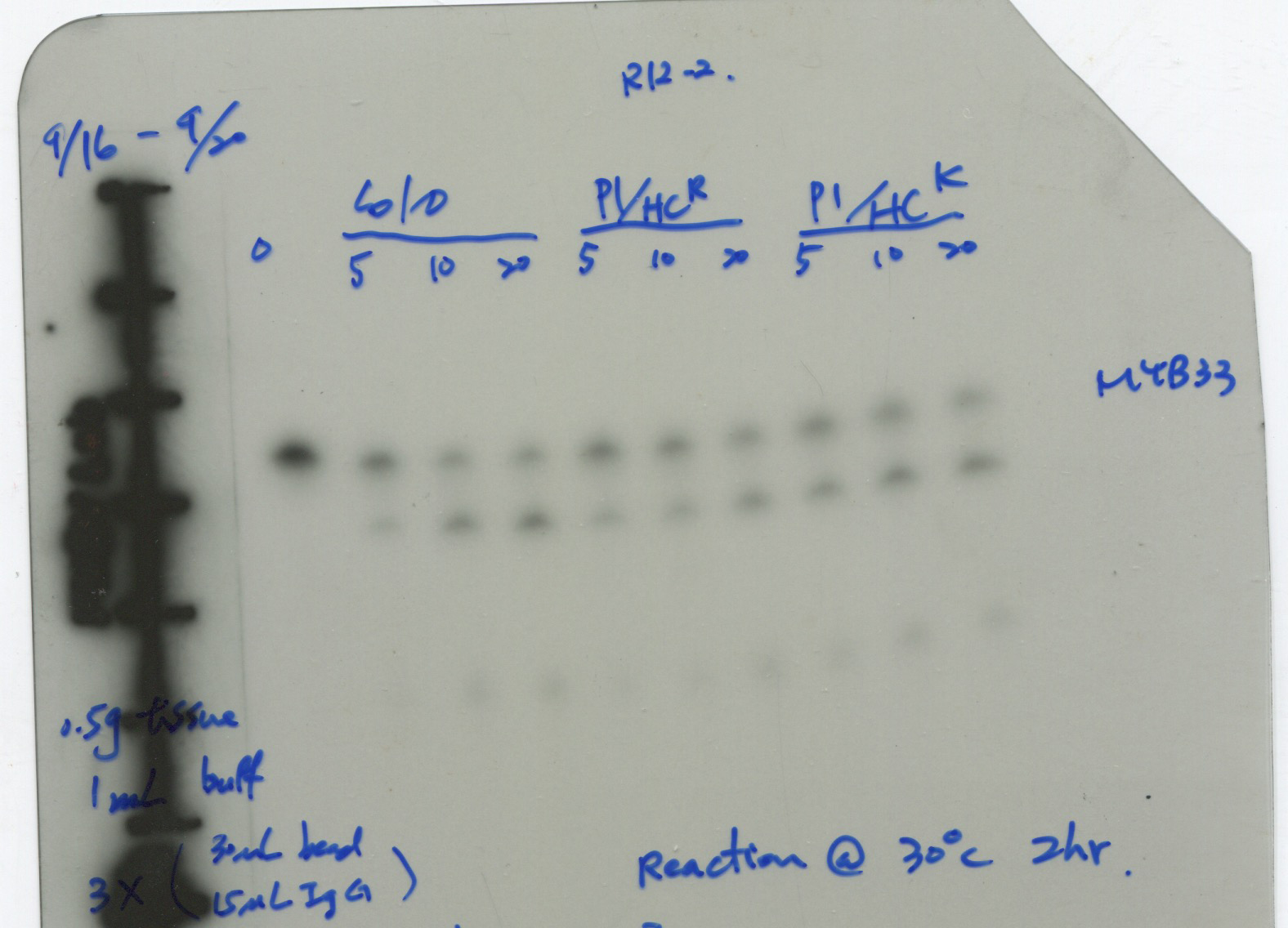

Supplement: Supplementary file 2 — Additional file 2. Supplementary Figures. [file 12985_2022_1956_MOESM2_ESM.zip › Additional file 2_Original images/Fig4c right.jpg]

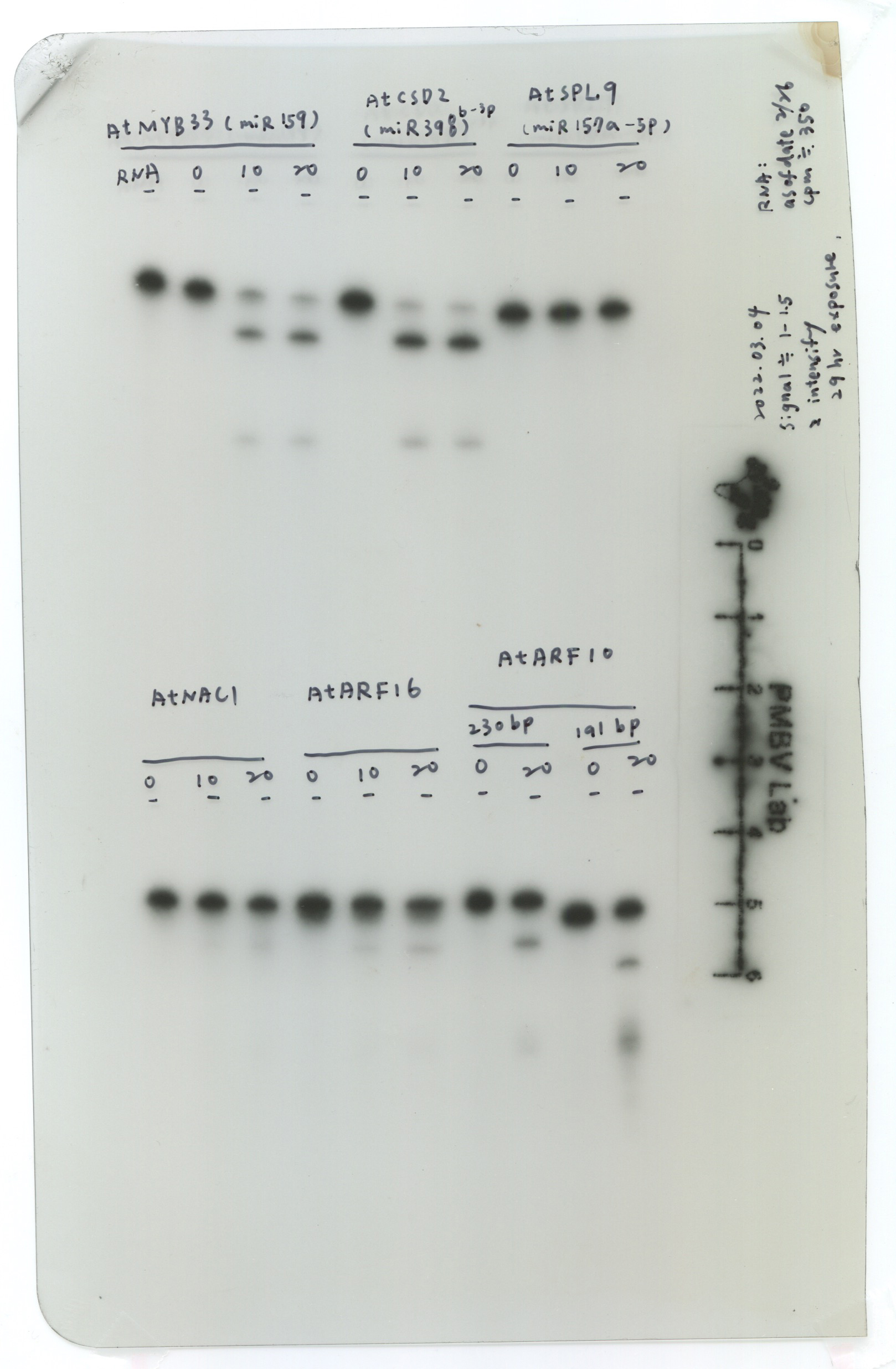

Supplement: Supplementary file 2 — Additional file 2. Supplementary Figures. [file 12985_2022_1956_MOESM2_ESM.zip › Additional file 2_Original images/Fig2g.jpg]

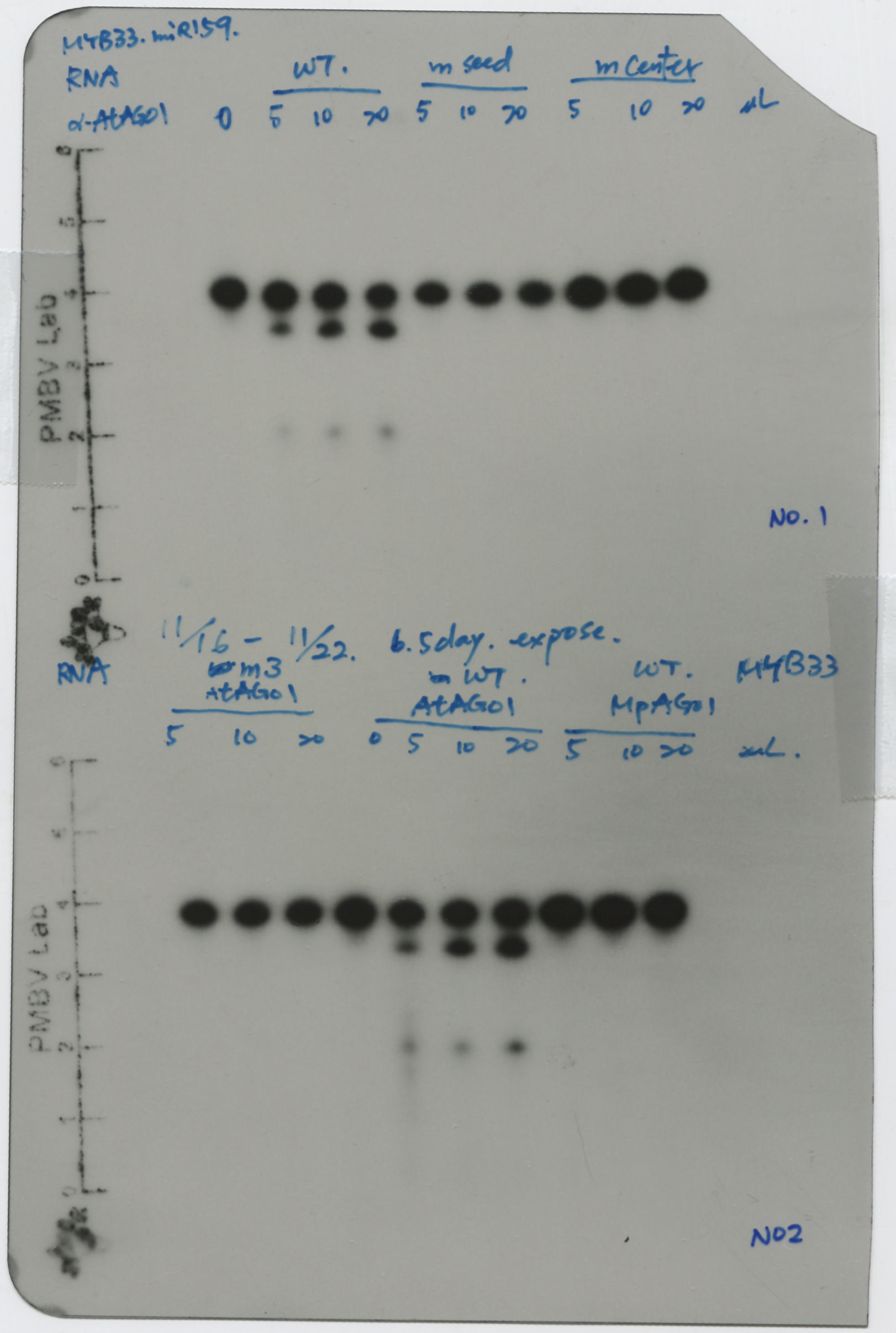

Supplement: Supplementary file 2 — Additional file 2. Supplementary Figures. [file 12985_2022_1956_MOESM2_ESM.zip › Additional file 2_Original images/Fig2f.jpg]

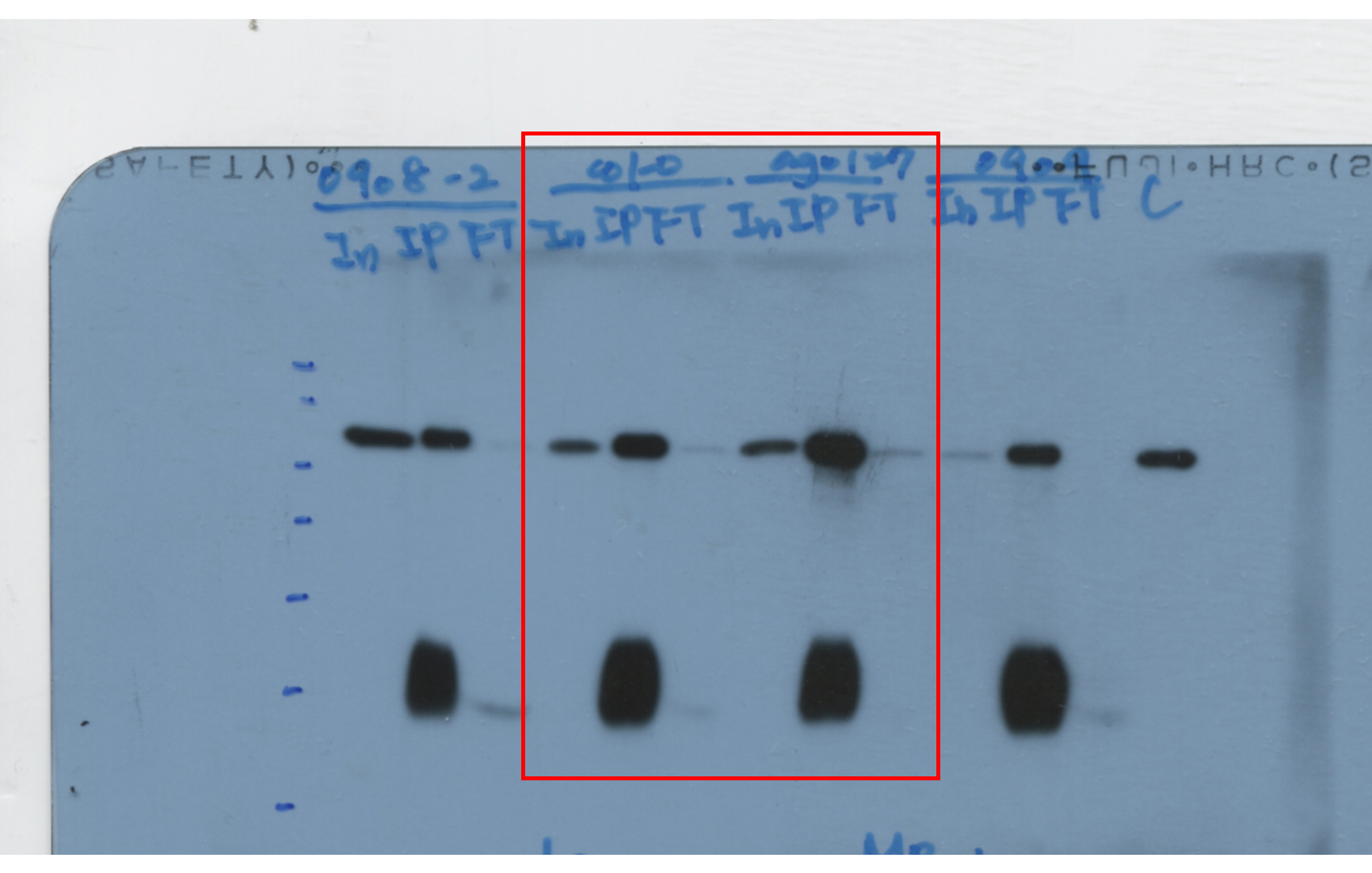

Supplement: Supplementary file 2 — Additional file 2. Supplementary Figures. [file 12985_2022_1956_MOESM2_ESM.zip › Additional file 2_Original images/Fig2c Label.jpg]

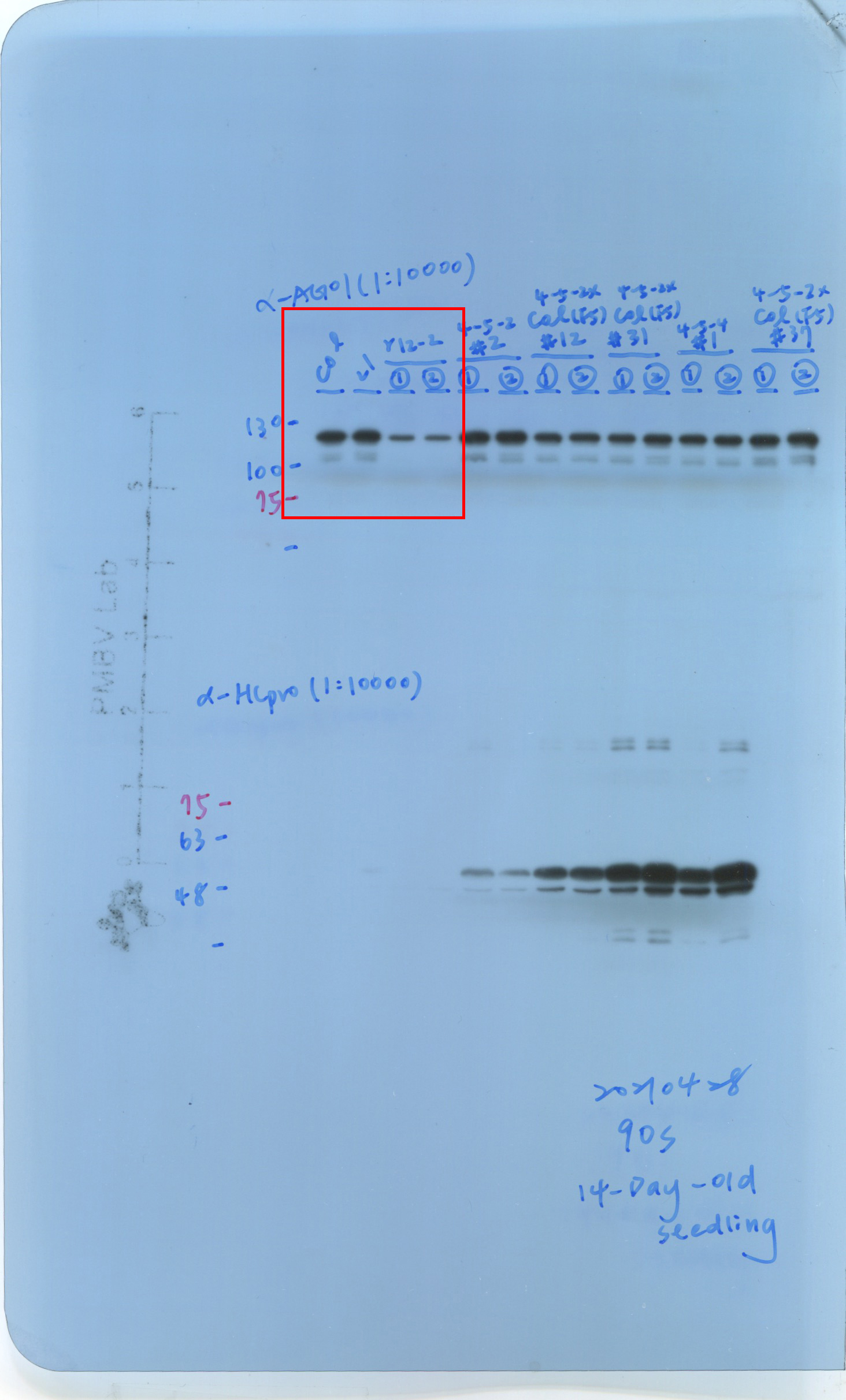

Supplement: Supplementary file 2 — Additional file 2. Supplementary Figures. [file 12985_2022_1956_MOESM2_ESM.zip › Additional file 2_Original images/Fig3c_AGO1 Label.jpg]

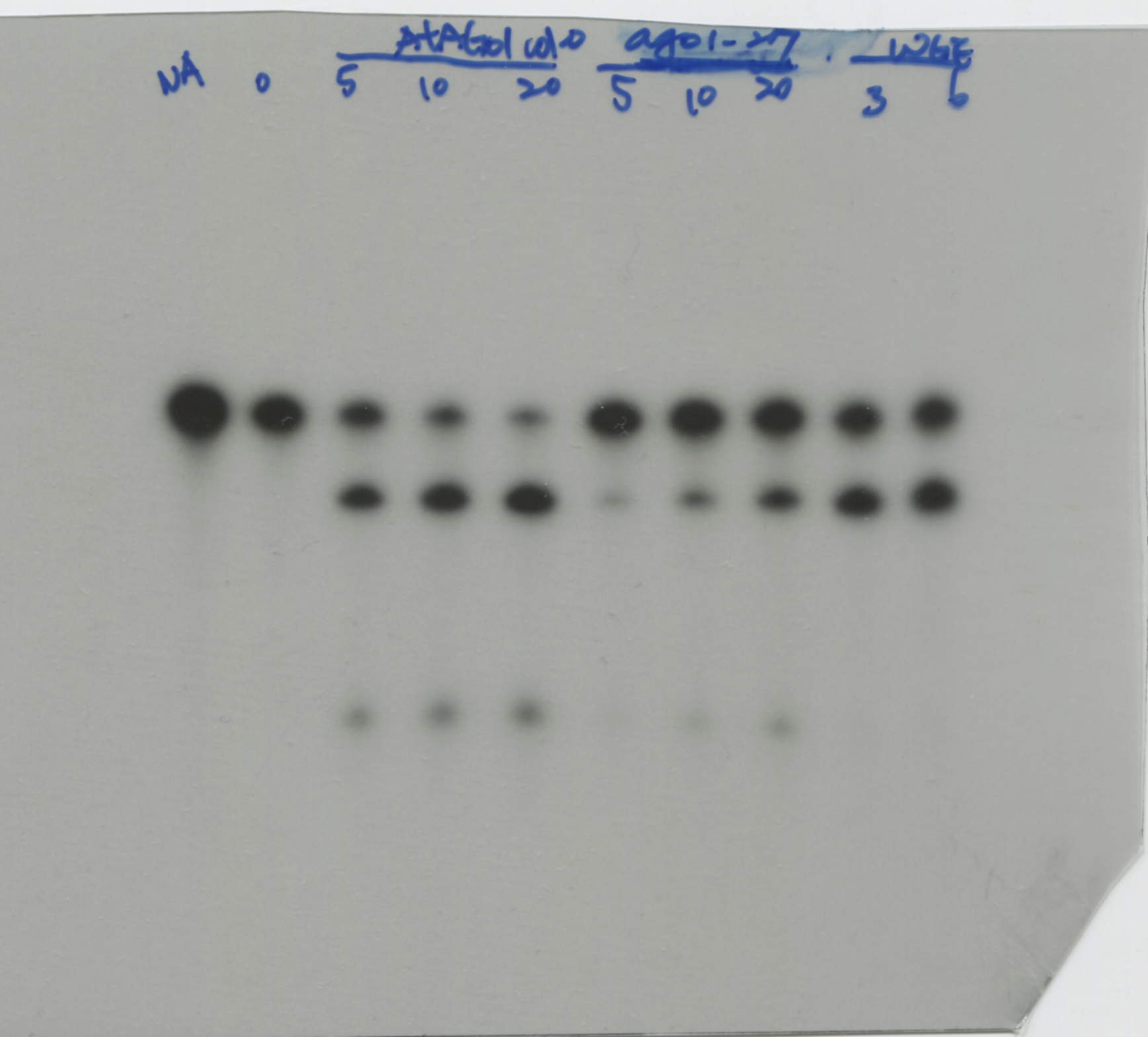

Supplement: Supplementary file 2 — Additional file 2. Supplementary Figures. [file 12985_2022_1956_MOESM2_ESM.zip › Additional file 2_Original images/Fig2b.jpg]
